# Health Disparities Among Hispanic Patients With Type 2 Diabetes in the United States: An Educational Workshop

**DOI:** 10.15766/mep_2374-8265.11622

**Published:** 2026-07-22

**Authors:** Yemmy Soler, Kaitlyn Pommells, Anita Laloo, Melissa Armas

**Affiliations:** 1 Assistant Professor, Department of Medical Education, Dr. Kiran C. Patel College of Allopathic Medicine at Nova Southeastern University; 2 Coordinator, Academic Medicine Writing Fellowship, Building the Next Generation of Academic Physicians; 3 Assistant Professor, Department of Medical Education, Dr. Kiran C. Patel College of Allopathic Medicine at Nova Southeastern University

**Keywords:** Health Disparities, Type 2 Diabetes, Social Determinants of Health, Case-Based Learning, Community Engagement, Hispanic Patients

## Abstract

**Introduction:**

Type 2 diabetes (T2D) is a major health concern for Hispanic populations in the United States. Hispanic patients experience a higher prevalence of T2D and more severe outcomes than non-Hispanic White populations, driven largely by systemic and structural factors such as limited access to care, insurance coverage gaps, language barriers, and social determinants of health. Access to culturally responsive and humility-based health care approaches remains limited, hindering patient-centered care.

**Methods:**

Using the 6-step Kern model, we designed, implemented, and evaluated a 60-minute interactive workshop to educate first- and second-year preclinical medical students about T2D in Hispanic patients. The workshop included a presentation, case-based discussions, a large-group debriefing, and pre- and postsession evaluations. Statistical analysis assessed changes in participant knowledge and confidence.

**Results:**

Following an initial virtual pilot session, the revised curriculum was delivered in person. Medical students completed pre- and postsession evaluations. The Mann-Whitney U test demonstrated significant increases in confidence across all learning objectives (*P* < .01). Knowledge scores also improved from pre- to postsession evaluations. Overall, 91% of participants rated the workshop as good or excellent. Feedback highlighted the value of content and structure, with suggestions to expand case discussions and introduce more varied activities.

**Discussion:**

The workshop effectively demonstrated an increase in medical student awareness of preventing and managing T2D among Hispanic patients. It emphasized cultural humility and provided a framework for exploring complex social, cultural, and structural factors. This workshop can be adapted for prehealth students, residents, or practicing physicians working with Hispanic patients.

## Educational Objectives

By the end of this activity, learners will be able to:

1. Describe health disparities among Hispanic patients with type 2 diabetes living in the United States.
2. Elucidate genetic susceptibility and metabolic factors that contribute to type 2 diabetes among Hispanic patients.
3. Describe cultural, socioeconomic, and lifestyle factors associated with the incidence of type 2 diabetes among Hispanic patients.
4. Illustrate through cases the need for more inclusive and tailored treatment options, preventive education, and health interventions that are necessary to improve patient outcomes.

## Introduction

Type 2 diabetes (T2D) is a major public health concern globally, affecting approximately 415 million individuals worldwide.^1^ In the United States (US), Hispanic patients are disproportionately affected by T2D, with a prevalence of 16.9% compared to 10.2% among non-Hispanic White individuals.^1,2^ In this article, the term “Hispanic” refers to a diverse group of individuals with ancestry from Spanish-speaking countries or cultures, including Mexico, Central America, South America, the Caribbean, and Spain. This term encompasses diversity in country of origin, race, socioeconomic status, language, and cultural practices.^3^ Therefore, T2D risk and outcomes may vary and should not be viewed as uniform across Hispanic populations.

Hispanic patients in the US have a 66% higher risk of developing T2D compared to non-Hispanic White patients. Studies indicate that Hispanic populations tend to develop T2D at younger ages and experience more severe outcomes, including higher rates of acute and chronic complications compared to non-Hispanic White individuals.^4–6^ Within the Hispanic community, T2D prevalence varies based on country of origin, with Mexicans, Puerto Ricans, Cubans, and Central/South Americans showing particularly high rates.^7,8^ Despite the growing number of Hispanic patients affected, T2D management among these populations remains suboptimal, with only 48% achieving adequate glycemic control.^9,10^ These disparities cannot be attributed solely to cultural or behavioral factors, but rather to the cumulative impact of structural inequities including limited access to health care, lack of insurance, socioeconomic disadvantage, and systemic barriers within the health care system.

The causes of these disparities are complex, including metabolic, genetic, environmental, socioeconomic, structural, and lifestyle factors.^11^ Metabolic factors, such as insulin resistance, obesity, beta cell dysfunction, dyslipidemia, and gut microbiome differences, contribute to increased susceptibility to T2D among Hispanic populations. Environmental and lifestyle factors, including dietary patterns, access to safe spaces for physical activity, and food insecurity, further shape disease risk. Social determinants shaped by structural inequities, such as medical mistrust, social isolation, community influence, and religion/spirituality, also affect health behaviors, treatment adherence, and engagement with health care services. While genetic studies have identified more than 100 genetic regions linked to T2D, they only explain a small portion of heritability. Most of these studies focus on European populations, leaving gaps in understanding T2D in Hispanic patients. However, recent studies have identified gene variants such as SLC16A11, HNF1A, and IGF2 that are more prevalent in Hispanic patients compared to non-Hispanic White patients. These variants influence T2D risk through mechanisms including altered insulin secretion, beta cell dysfunction, and impaired glucose metabolism.^12–17^ Understanding population-specific genetic factors is crucial for identifying individuals at high risk and developing effective prevention and treatment strategies tailored to diverse groups.

In addition, Hispanic patients with T2D face significant barriers to effective care due to structural and systemic inequities. These include limited access to insurance, language barriers, financial constraints, and lower access to structurally informed and patient-centered care models. These issues, coupled with systemic health care disparities, contribute to poorer diabetes management and outcomes. For example, from the Hispanic Community Health Study/Study of Latinos, it was found that nearly half of Hispanic patients with diabetes were uninsured.^10^ Structural issues within the health care system, such as high treatment costs and limited access to culturally responsive care, further complicate the situation. Culturally competent care refers to the ability of health care providers and institutions to deliver care that is respectful of and responsive to patients’ cultural health beliefs, practices, and language needs. When patients do not receive culturally responsive care, it can lead to misunderstandings, mistrust, reduced treatment adherence, and ultimately poorer health outcomes. This is especially critical for chronic conditions like T2D, where long-term management depends heavily on effective communication, patient education, and trust in the health care system.^18–20^

Given the diversity within Hispanic populations and the structural barriers that shape access to care, this educational workshop adapts a framework of cultural humility. It emphasizes lifelong learning, self-reflection, and recognition of power imbalances within health care systems while acknowledging the diversity across Hispanic communities. This approach is well suited to chronic disease management, such as T2D. By explicitly considering social factors, the workshop encourages learners to examine how these determinants can influence diabetes management and patient engagement in real-world clinical settings.

Building on this framework, medical education must incorporate training that addresses systemic inequities and promotes cultural humility to improve care for this high-risk population. However, despite the documented disparities, there remains a notable gap in medical education concerning the unique challenges and needs of Hispanic patients with T2D. A review of published curricula in *MedEdPORTAL* and related educational resources reveals that most existing diabetes educational workshops focus on general disease mechanisms or basic patient education without integrating sociocultural, economic, genetic, and epidemiologic considerations. For example, workshops such as Diabetes Mellitus: Diagnosis, Prevention and Goals of Therapy and Finding the Sweet Spot: An Interactive Workshop on Diabetes Management in Older Adults provide overviews of pathophysiology and management but lack content on cultural competence or minority-specific barriers to care.^21,22^ Another *MedEdPORTAL* workshop addressing endocrine diseases in Hispanic populations titled Medical Spanish Endocrinology Educational Module focuses primarily on addressing the need for improved Spanish language and communication skills among medical students, but it does not directly address broader pathophysiological, biological, systemic, or structural factors of the disease.^23^

This workshop was created to address this gap by educating preclinical medical students about the intersection of T2D and health disparities among Hispanic patients in the US. The workshop represents a novel contribution by integrating clinical epidemiology, genetics, and a cultural humility framework into a single cohesive learning experience centered on the systemic causes of inequity. By equipping future physicians with this knowledge, we aim to foster more inclusive, empathetic, and effective care.

## Methods

We developed an interactive, 60-minute educational workshop using the 6-step Kern Model of Curriculum Development.^24^

### Step 1: Problem Identification and General Needs Assessment

We conducted a literature review and assessed current curricula and medical training on T2D among Hispanic patients in the US. Based on our search, we identified major health disparities affecting Hispanic patients that are insufficiently addressed in medical education, particularly with respect to structurally imposed barriers and systemic inequities within the health care system.^1,5,7,10,20^

### Step 2: Targeted Needs Assessment

We reviewed knowledge gaps, clinical skills, and attitudes of first- and second-year medical students regarding T2D in Hispanic patients. We also gathered input from faculty and content experts to ensure that the curriculum addressed learners’ educational needs and aligned with clinical practice relevance. An initial virtual pilot session of the educational intervention was delivered at a national conference and functioned as an informal needs assessment. Due to low participation, small-group breakout discussions were not feasible, and the session was conducted as a single-group discussion. Pre- and postsession evaluations were administered. However, response rates were low and insufficient for meaningful quantitative analysis. Instead, qualitative feedback, participant questions, facilitator observations, and overall learner engagement from the first session were used to identify gaps in content, clarity, and structure. This feedback informed revisions to the workshop described here, including refinement of learning objectives, expansion of content related to structural and systemic determinants of health, and enhancement of case-based discussion prompts. The revised curriculum was then implemented during a second, in-person session, which serves as the primary focus of the results reported in this study.

### Step 3: Goals and Objectives

We created the learning objectives with input from faculty and content experts using Bloom's Taxonomy.^25^ These learning objectives focused on increasing knowledge about T2D disparities affecting Hispanic patients, understanding structural barriers, and applying cultural humility–based approaches in patient care. The objectives were designed to target both knowledge acquisition and learner confidence addressing these topics.

### Step 4: Educational Strategies

The chosen educational strategies included a Microsoft PowerPoint presentation, reflective questions, case-based discussions, and large-group debriefings. These strategies were chosen to promote engagement, critical thinking, and application of knowledge to real-world scenarios.

### Step 5: Implementation

Following revisions informed by the virtual pilot session, the finalized curriculum was implemented during a second, in-person educational session. This in-person session took place in an auditorium classroom at Nova Southeastern University (NSU), which allowed for more structured small-group discussions, greater learner engagement, and robust facilitation of case-based learning. Participants included first- and second-year preclinical medical students. No prior medical knowledge specific to T2D pathophysiology, glucose regulation, or insulin resistance was required. Prior experience with diabetes care or working with Hispanic patients was not necessary. Materials required for implementation included internet access, a computer, a projector, and audio equipment for PowerPoint presentation delivery. Participants used personal electronic devices including smartphones, tablets, and computers to scan the QR codes and complete the pre- and postsession evaluations.

During the 60-minute workshop, participants completed the presession evaluation (Appendix A), which assessed baseline knowledge of T2D in Hispanic patients and confidence in achieving the learning objectives, followed by a PowerPoint presentation. The PowerPoint presentation (Appendix C) was developed by 3 faculty members (Yemmy Soler, Melissa Armas, and Anita Laloo) from Dr. Kiran C. Patel College of Allopathic Medicine at NSU with expertise in science, health, and medical education. One faculty member (Yemmy Soler) facilitated both virtual and in-person sessions. The presentation began with an overview of T2D disparities among Hispanic populations, including difference in prevalence, complications, preventative care, and structural barriers to care. It then explored genetic, biological, and metabolic contributions to T2D risks, incorporating findings from recent genomic and epidemiologic research. Next, we presented 3 case studies with a set of guiding questions designed to facilitate group discussion, prompt critical thinking, encourage the exploration of key issues and implications, and promote the proposal of solutions. Participants were split into 16 small groups with an average of 3–4 students per group (range 2–5 students). They were given 10–15 minutes to read the scenario, discuss the case, and answer the questions. After finishing the case discussions, a large group debriefing took place where we went over and summarized the key take-home messages such as the socioeconomic, cultural, and lifestyle factors that can influence the incidence of T2D among Hispanic patients. Essential talking points for small-group discussions can be found in both the speaker notes in the PowerPoint (see Appendix C) and the facilitator guide (Appendix D). Afterward, we asked participants to complete the postsession evaluation (Appendix B). Time (5–10 minutes) was allotted for questions and completion of postsession evaluation.

### Step 6: Evaluation and Feedback

We administered pre- and postsession evaluations to assess participants’ change in knowledge, confidence, satisfaction, and educational effectiveness of the workshop. We assessed participants’ confidence in achieving the objectives using a Likert scale and participants’ change in knowledge through multiple-choice questions. We also asked participants postsession evaluation open-text questions: “What did you like about the workshop?” “What suggestions do you have to improve this workshop?” and “How would you implement what you learned in this workshop in the future?” Data was analyzed using Prism GraphPad version 10.4.1. The Mann-Whitney U test was used to compare confidence levels of participants before and after the session,^26,27^ whereas Fisher's exact test was used to determine if there was an improvement in knowledge based on correct responses before and after the session.^26–29^

We collected demographic data with the primary purpose of understanding the general characteristics of learner groups, offering context for the evaluation results. Because the pre- and postsession evaluations were not linked at the individual level, we could not analyze how these demographic variables influenced changes in knowledge or confidence. However, reporting general learner demographics helps to understand the audience, assess applicability, and contextualize the findings achieved by the workshop.

We received approval for this project from the Institutional Review Board at NSU (Protocol No. 2424-257).

## Results

This workshop was implemented twice: once as a virtual session at the 2024 BNGAP National Conference via Zoom, and once in-person at NSU. All results reported are derived from the NSU implementation.

Fifty-two first- and second-year preclinical medical students participated, of whom 48 completed the presession evaluation and 45 completed the postsession evaluation (93% response rate). Of the 48 medical students who completed the presession evaluation and completed the optional race/ethnicity question, the results are as follows: 62.5% White; 27.1% Hispanic; 18.8% Asian; 6.3% Black or African American; 4.2% Middle Eastern or North American; and 2.1% Native American, Alaskan, or Pacific Islander. Participants self-assessed their confidence in achieving the workshop's learning objectives on a scale from 0 (no confidence) to 4 (complete confidence). Response analysis revealed a significant increase (*P* < .01) in confidence for all learning objectives (Table 1). Multiple-choice questions designed to assess participants’ baseline knowledge and their knowledge acquisition after the session showed that scores on postsession evaluations were significantly greater than presession evaluations (Fisher's exact test, *P* < .01), indicating an effective enhancement in participants’ awareness of T2D disparities among Hispanic populations (Table 2).

**Table 1.**
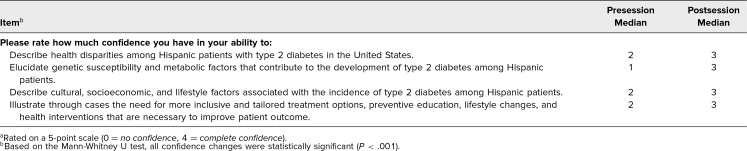
Participants' Self-Assessed Confidence in Addressing Health Disparities Among Hispanic Patients with Type 2 Diabetes in the United States^a^ (*N* = 45)

**Table 2.**
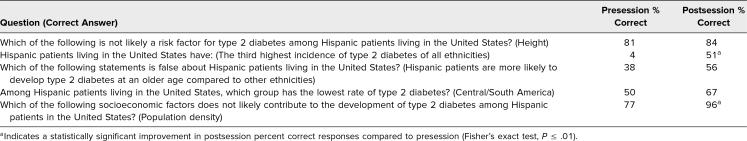
Percent Correct on Pre- and Postsession Knowledge Questions Before and After the Workshop (*N* = 45)

Upon completion of the workshop, 47% of participants rated the module as excellent, 44% as good, and 9% as fair. Suggestions for improvement included “shorter vignettes and more discussion time” and “more variety of engaging activities.” Qualitative feedback regarding the module's strength focused on case discussion, content, interaction, organization, and statistics. Selective examples of feedback include “I like the cases and how they were realistic,” “I liked that the scenarios were fleshed out so there was a lot to discuss and the open-ended questions allowed us to explore multiple areas of handling chronic conditions like diabetes,” and “I liked the awareness to Hispanic populations in regards to Type 2 Diabetes and the challenges that may be faced in clinical practice.”

## Discussion

This educational workshop successfully increased medical students’ awareness of the challenges faced by Hispanic patients with T2D in the US. Through structured discussions and case-based learning, this session allowed participants to explore the systemic, structural, and social determinants of health that contribute to the higher prevalence of T2D among Hispanic communities.

Our results revealed a statistically significant improvement in participants’ confidence in understanding and addressing health disparities among Hispanic patients with T2D (*P* < .01). These results align with the workshop's goal of enhancing medical students’ knowledge and awareness about the distinct risks faced by Hispanic populations. The increase in confidence among students suggests that the workshop successfully conveyed essential knowledge. Additionally, the descriptive multiple-choice questions included in the pre- and postsession evaluations demonstrated an improvement in knowledge acquisition. The increase in the average scores between the pre- and posttests indicates that the workshop effectively bridged knowledge gaps regarding T2D in Hispanic patients. This supports the premise that targeted educational initiatives incorporating cultural humility can improve the understanding of specific health disparities and guide more effective patient care in diverse populations.

In general, the workshop was well received, with 91% of participants rating it as “excellent” or “good.” Nevertheless, there were suggestions for improvement. Some participants recommended modifying the case studies to better reflect the diverse experiences within the Hispanic community, including different nationalities (eg, Mexicans, Puerto Ricans, Cubans), as diabetes rates vary significantly among them. Others suggested incorporating more activities that engage participants in real-life problem-solving scenarios to deepen their understanding of the cultural and psychological aspects of diabetes care in Hispanic populations.

Despite the positive outcomes, this workshop has several limitations. The small, nonrandom sample may not reflect broader medical student populations. As such, findings may not be generalizable. Only the immediate outcomes of the workshop were assessed. Long-term knowledge retention was not measured, which is critical for determining the true impact of educational interventions. Future iterations of this module could include follow-up assessments as learners begin clinical rotations to determine not only the durability of knowledge but also the ability to apply cultural humility–based approaches to Hispanic patients with T2D in real-world clinical settings. Results are based on a single in-person implementation, which may limit generalizability. Future studies should evaluate the intervention across multiple settings and formats. We also recognize that differences in professional roles and prior exposure to diabetes education could introduce selection bias, potentially affecting baseline knowledge and confidence scores. Finally, while demographic factors such as professional role, training level, and prior exposure to diabetes management may influence baseline knowledge, our evaluation collected self-reported confidence to account for these differences.

Nevertheless, the results of this workshop suggest that integrating a cultural humility–based approach into medical education, especially in areas related to chronic conditions like T2D, is essential. By emphasizing cultural humility, health care professionals can better support preventive measures and early intervention strategies, thereby improving long-term health outcomes. Future efforts should focus on (1) continuing to educate about specific health disparities, as well as genetic, metabolic, and cultural factors influencing the risk of T2D in diverse populations; (2) broadening the range of patient cases and experiences discussed in training, including more variation based on nationality, socioeconomic status, and personal challenges; and (3) implementing similar workshops regularly across medical institutions to ensure that a larger number of students and health professionals can address health care disparities in Hispanic communities.

This workshop represents a critical step toward enhancing medical education in the US. By integrating culturally sensitive education and real-world case discussions, the workshop equipped medical students with the knowledge and skills to better manage and prevent T2D in Hispanic patients. The improvements in participants’ knowledge and confidence are promising, and the feedback gathered from the students can guide future iterations to make these educational tools even more effective. As the burden of T2D continues to rise, particularly among minority populations, it is essential that health care providers receive training that emphasizes the importance of cultural humility–based care, personalized interventions, and a comprehensive understanding of the structural and systemic factors contributing to health disparities in Hispanic populations.

## Appendices


Presession Evaluation.docxPostsession Evaluation.docxPresentation.pptxFacilitator Guide.docx
*All appendices are peer reviewed as integral parts of the Original Publication.*
