# Supplementary material for: Health Disparities Among Hispanic Patients With Type 2 Diabetes in the United States: An Educational Workshop
Source: MedEdPORTAL. 2026 Jul 22;22:11622. doi: 10.15766/mep_2374-8265.11622 (PMC13388404; doi:10.15766/mep_2374-8265.11622)
Supplement: Supplementary file 1 — Presession Evaluation.docxPostsession Evaluation.docxPresentation.pptxFacilitator Guide.docx [file mep_2374-8265.11622-s001.zip › C. Presentation.pptx]

## Slide 1
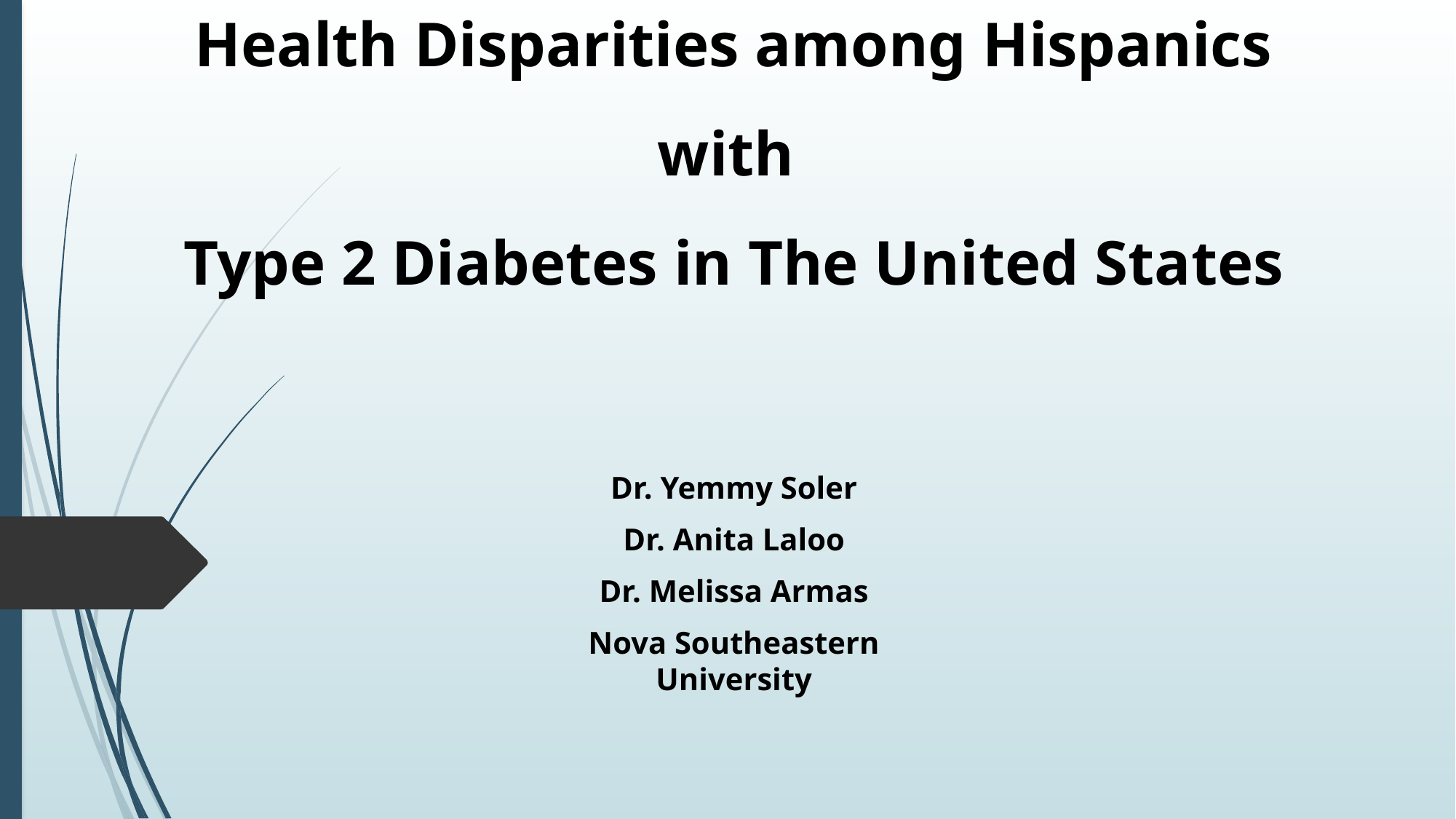

# Health Disparities among Hispanics with Type 2 Diabetes in The United States
Dr. Yemmy Soler
Dr. Anita Laloo
Dr. Melissa Armas
Nova Southeastern University

## Slide 2
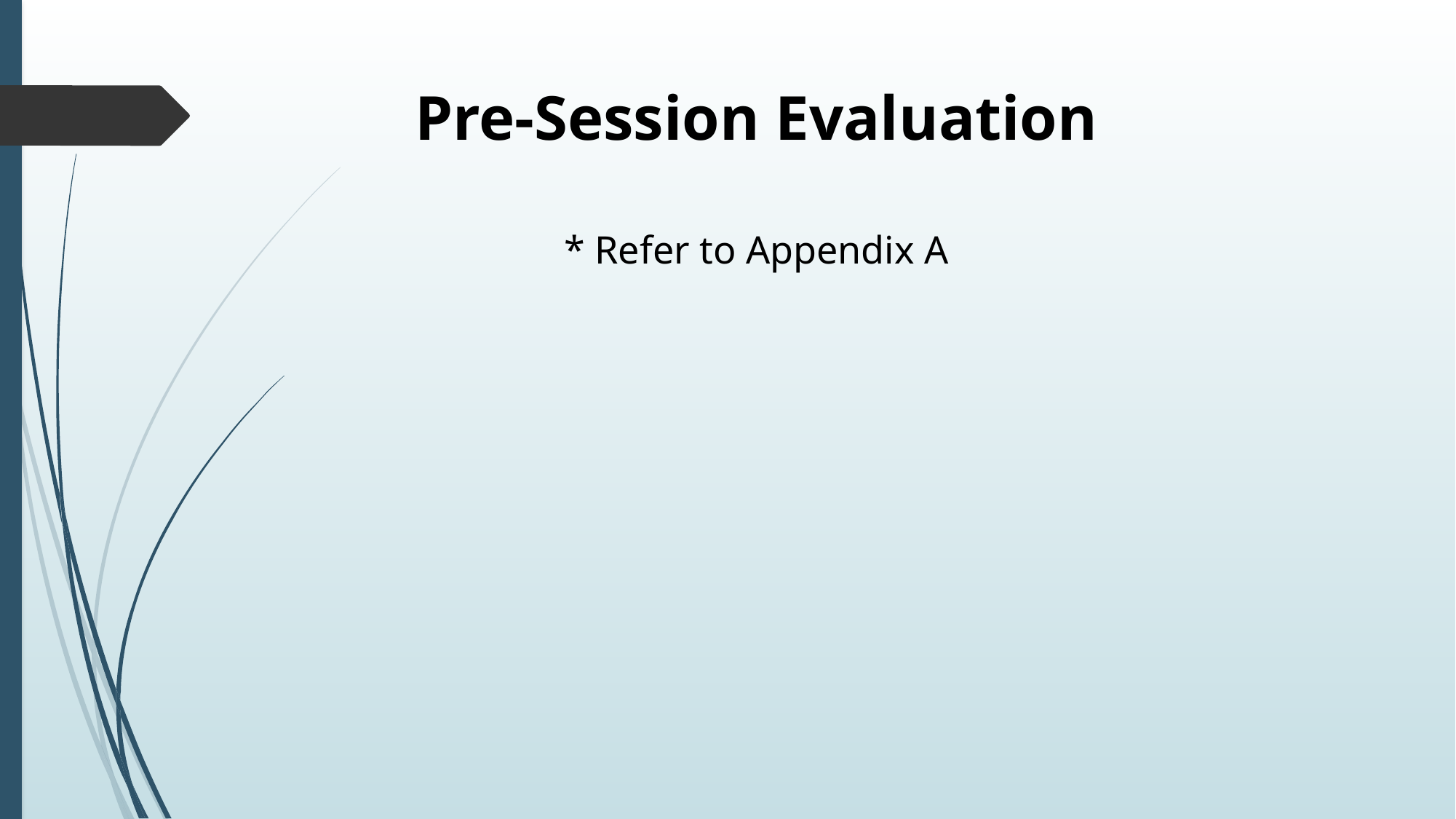

# Pre-Session Evaluation
* Refer to Appendix A

## Slide 3
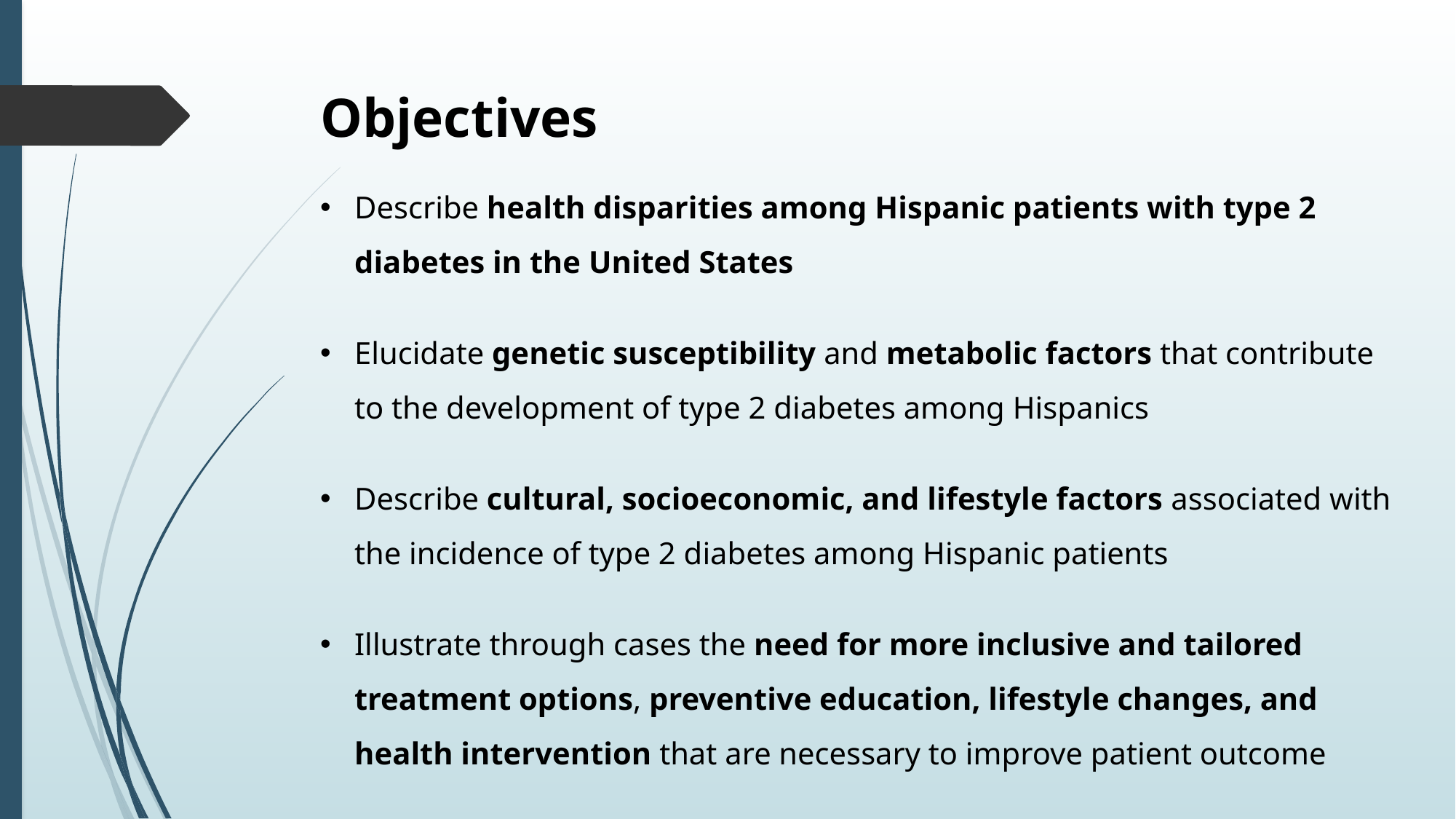

# Objectives
Describe health disparities among Hispanic patients with type 2 diabetes in the United States
Elucidate genetic susceptibility and metabolic factors that contribute to the development of type 2 diabetes among Hispanics
Describe cultural, socioeconomic, and lifestyle factors associated with the incidence of type 2 diabetes among Hispanic patients
Illustrate through cases the need for more inclusive and tailored treatment options, preventive education, lifestyle changes, and health intervention that are necessary to improve patient outcome

## Slide 4
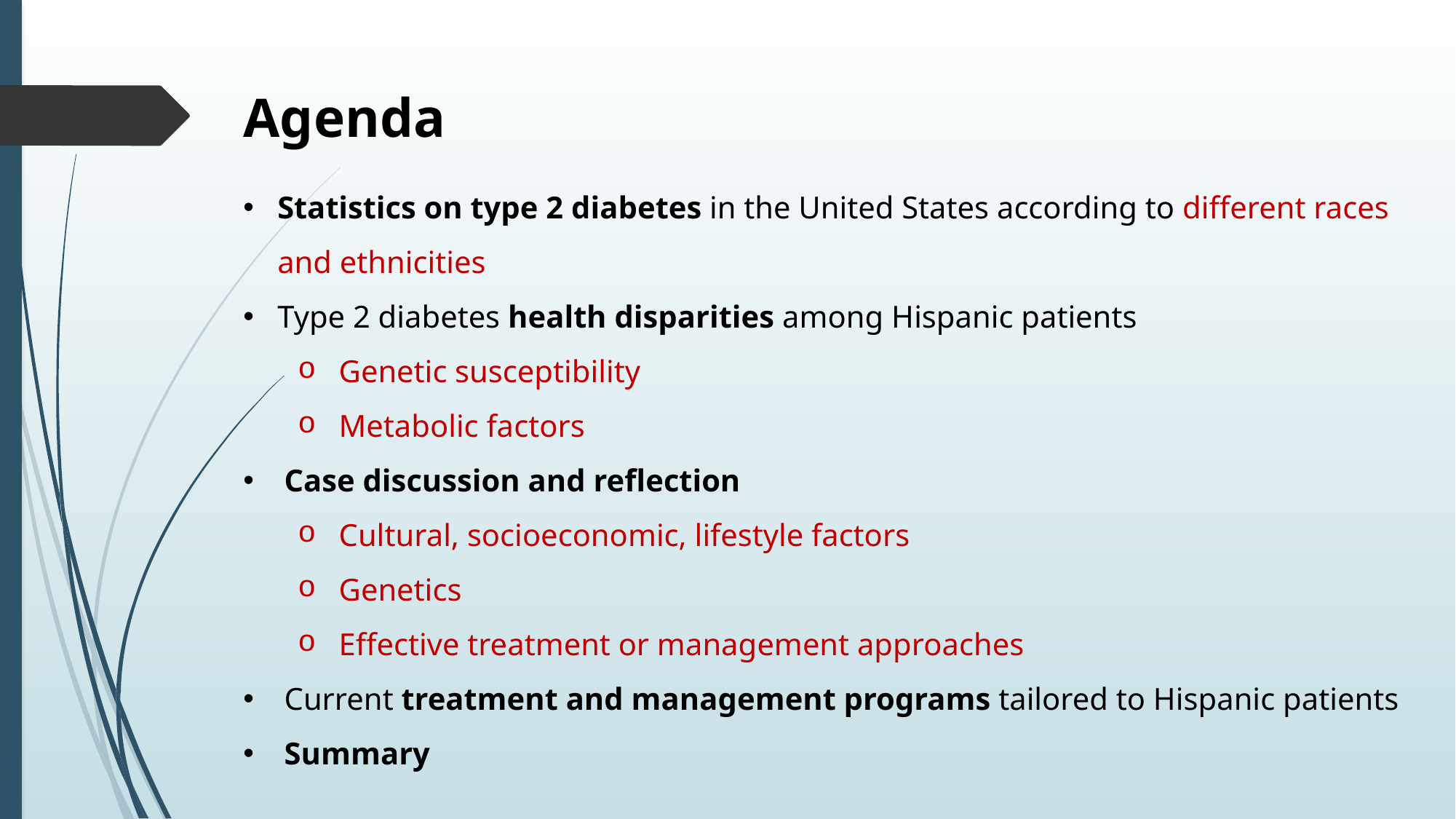

# Agenda
Statistics on type 2 diabetes in the United States according to different races and ethnicities
Type 2 diabetes health disparities among Hispanic patients
Genetic susceptibility
Metabolic factors
Case discussion and reflection
Cultural, socioeconomic, lifestyle factors
Genetics
Effective treatment or management approaches
Current treatment and management programs tailored to Hispanic patients
Summary

## Slide 5
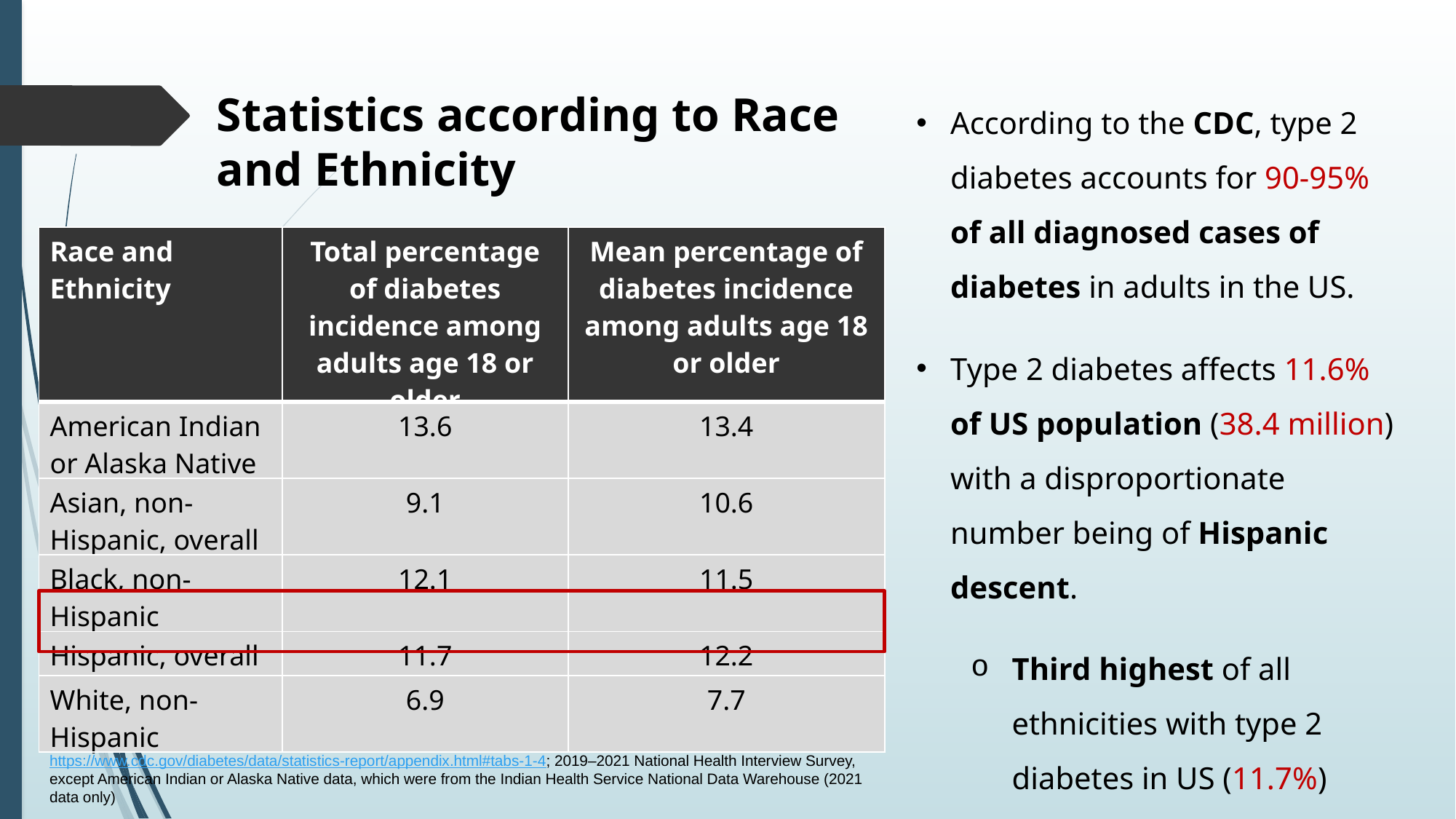

# Statistics according to Race and Ethnicity
According to the CDC, type 2 diabetes accounts for 90-95% of all diagnosed cases of diabetes in adults in the US.
Type 2 diabetes affects 11.6% of US population (38.4 million) with a disproportionate number being of Hispanic descent.
Third highest of all ethnicities with type 2 diabetes in US (11.7%)
| Race and Ethnicity | Total percentage of diabetes incidence among adults age 18 or older | Mean percentage of diabetes incidence among adults age 18 or older |
| --- | --- | --- |
| American Indian or Alaska Native | 13.6 | 13.4 |
| Asian, non-Hispanic, overall | 9.1 | 10.6 |
| Black, non-Hispanic | 12.1 | 11.5 |
| Hispanic, overall | 11.7 | 12.2 |
| White, non-Hispanic | 6.9 | 7.7 |
Data Source: Centers for Disease Control and Prevention; https://www.cdc.gov/diabetes/data/statistics-report/appendix.html#tabs-1-4; 2019–2021 National Health Interview Survey, except American Indian or Alaska Native data, which were from the Indian Health Service National Data Warehouse (2021 data only)

## Slide 6
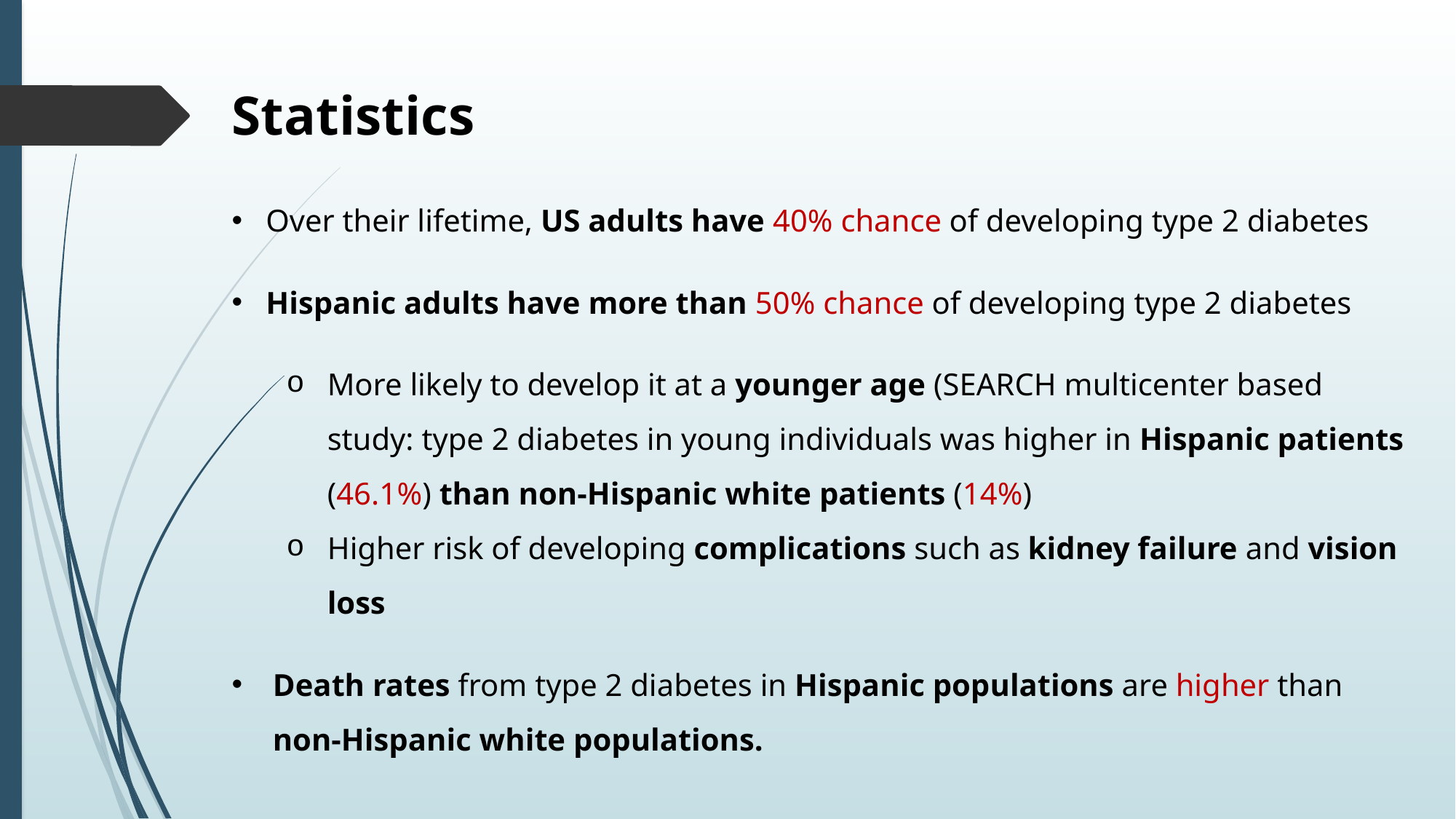

# Statistics
Over their lifetime, US adults have 40% chance of developing type 2 diabetes
Hispanic adults have more than 50% chance of developing type 2 diabetes
More likely to develop it at a younger age (SEARCH multicenter based study: type 2 diabetes in young individuals was higher in Hispanic patients (46.1%) than non-Hispanic white patients (14%)
Higher risk of developing complications such as kidney failure and vision loss
Death rates from type 2 diabetes in Hispanic populations are higher than non-Hispanic white populations.

## Slide 7
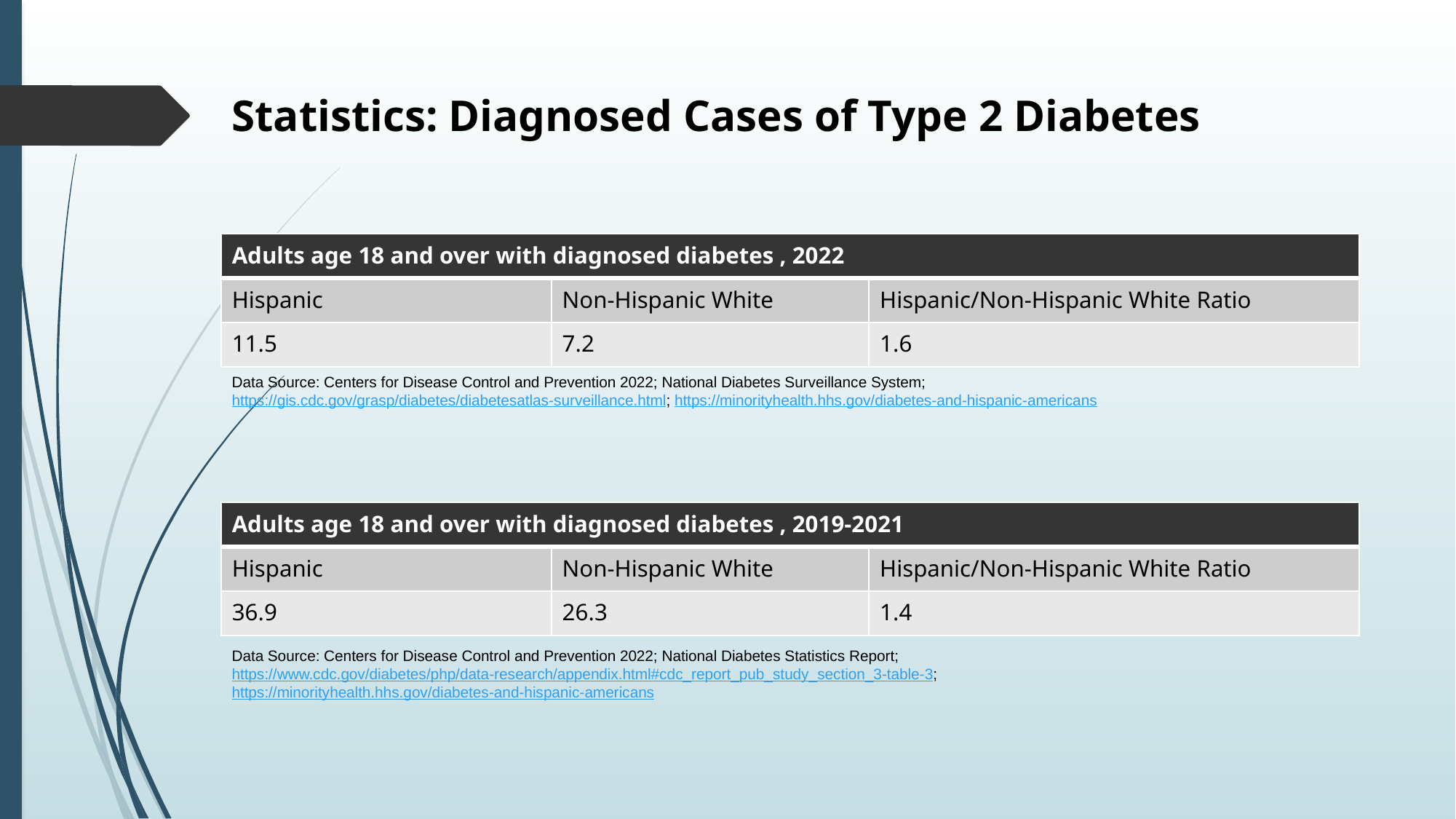

# Statistics: Diagnosed Cases of Type 2 Diabetes
| Adults age 18 and over with diagnosed diabetes , 2022 | | |
| --- | --- | --- |
| Hispanic | Non-Hispanic White | Hispanic/Non-Hispanic White Ratio |
| 11.5 | 7.2 | 1.6 |
Data Source: Centers for Disease Control and Prevention 2022; National Diabetes Surveillance System; https://gis.cdc.gov/grasp/diabetes/diabetesatlas-surveillance.html; https://minorityhealth.hhs.gov/diabetes-and-hispanic-americans
| Adults age 18 and over with diagnosed diabetes , 2019-2021 | | |
| --- | --- | --- |
| Hispanic | Non-Hispanic White | Hispanic/Non-Hispanic White Ratio |
| 36.9 | 26.3 | 1.4 |
Data Source: Centers for Disease Control and Prevention 2022; National Diabetes Statistics Report; https://www.cdc.gov/diabetes/php/data-research/appendix.html#cdc_report_pub_study_section_3-table-3; https://minorityhealth.hhs.gov/diabetes-and-hispanic-americans

## Slide 8
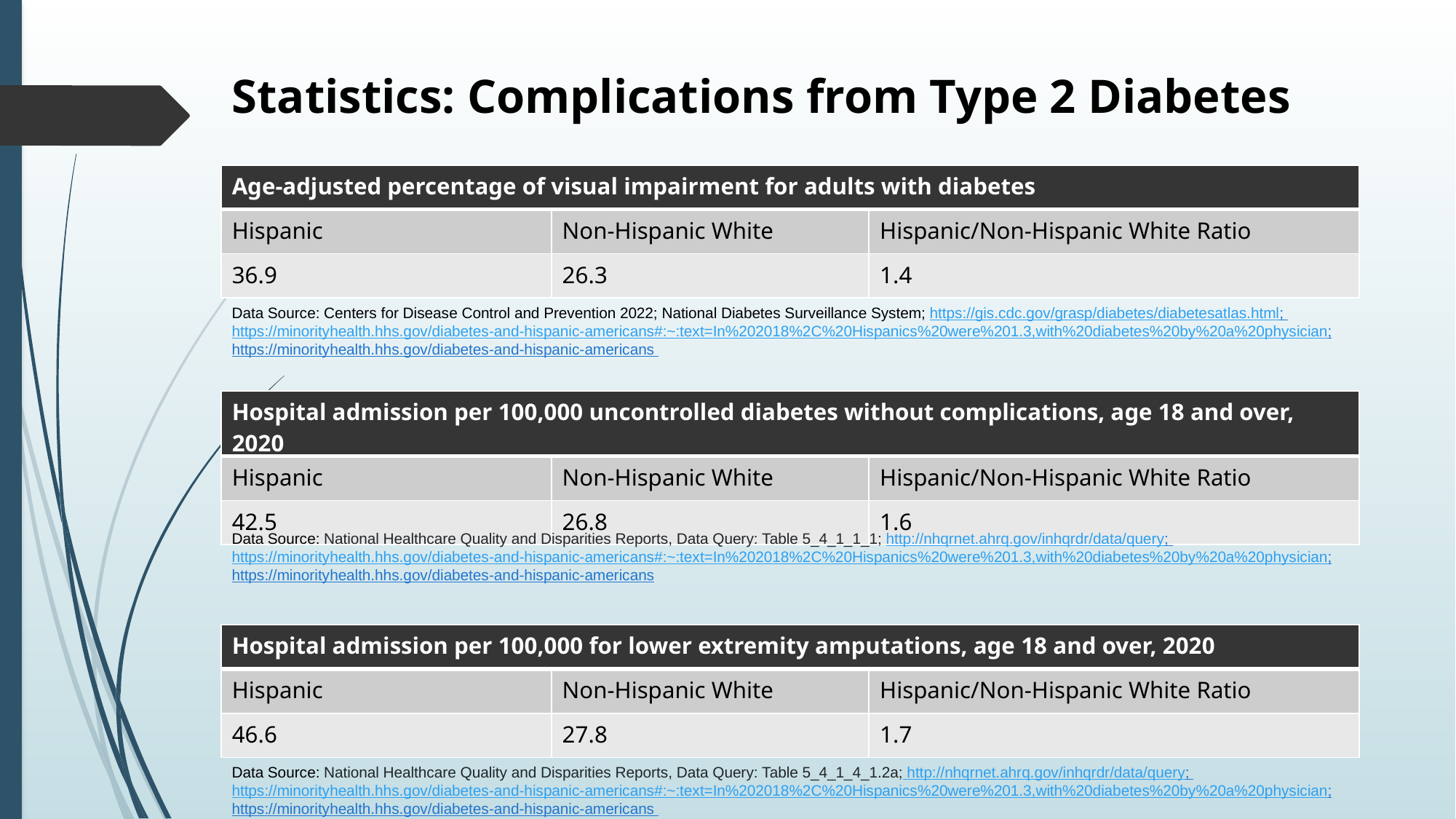

# Statistics: Complications from Type 2 Diabetes
| Age-adjusted percentage of visual impairment for adults with diabetes | | |
| --- | --- | --- |
| Hispanic | Non-Hispanic White | Hispanic/Non-Hispanic White Ratio |
| 36.9 | 26.3 | 1.4 |
Data Source: Centers for Disease Control and Prevention 2022; National Diabetes Surveillance System; https://gis.cdc.gov/grasp/diabetes/diabetesatlas.html; https://minorityhealth.hhs.gov/diabetes-and-hispanic-americans#:~:text=In%202018%2C%20Hispanics%20were%201.3,with%20diabetes%20by%20a%20physician; https://minorityhealth.hhs.gov/diabetes-and-hispanic-americans
| Hospital admission per 100,000 uncontrolled diabetes without complications, age 18 and over, 2020 | | |
| --- | --- | --- |
| Hispanic | Non-Hispanic White | Hispanic/Non-Hispanic White Ratio |
| 42.5 | 26.8 | 1.6 |
Data Source: National Healthcare Quality and Disparities Reports, Data Query: Table 5_4_1_1_1; http://nhqrnet.ahrq.gov/inhqrdr/data/query; https://minorityhealth.hhs.gov/diabetes-and-hispanic-americans#:~:text=In%202018%2C%20Hispanics%20were%201.3,with%20diabetes%20by%20a%20physician; https://minorityhealth.hhs.gov/diabetes-and-hispanic-americans
| Hospital admission per 100,000 for lower extremity amputations, age 18 and over, 2020 | | |
| --- | --- | --- |
| Hispanic | Non-Hispanic White | Hispanic/Non-Hispanic White Ratio |
| 46.6 | 27.8 | 1.7 |
Data Source: National Healthcare Quality and Disparities Reports, Data Query: Table 5_4_1_4_1.2a; http://nhqrnet.ahrq.gov/inhqrdr/data/query; https://minorityhealth.hhs.gov/diabetes-and-hispanic-americans#:~:text=In%202018%2C%20Hispanics%20were%201.3,with%20diabetes%20by%20a%20physician; https://minorityhealth.hhs.gov/diabetes-and-hispanic-americans

## Slide 9
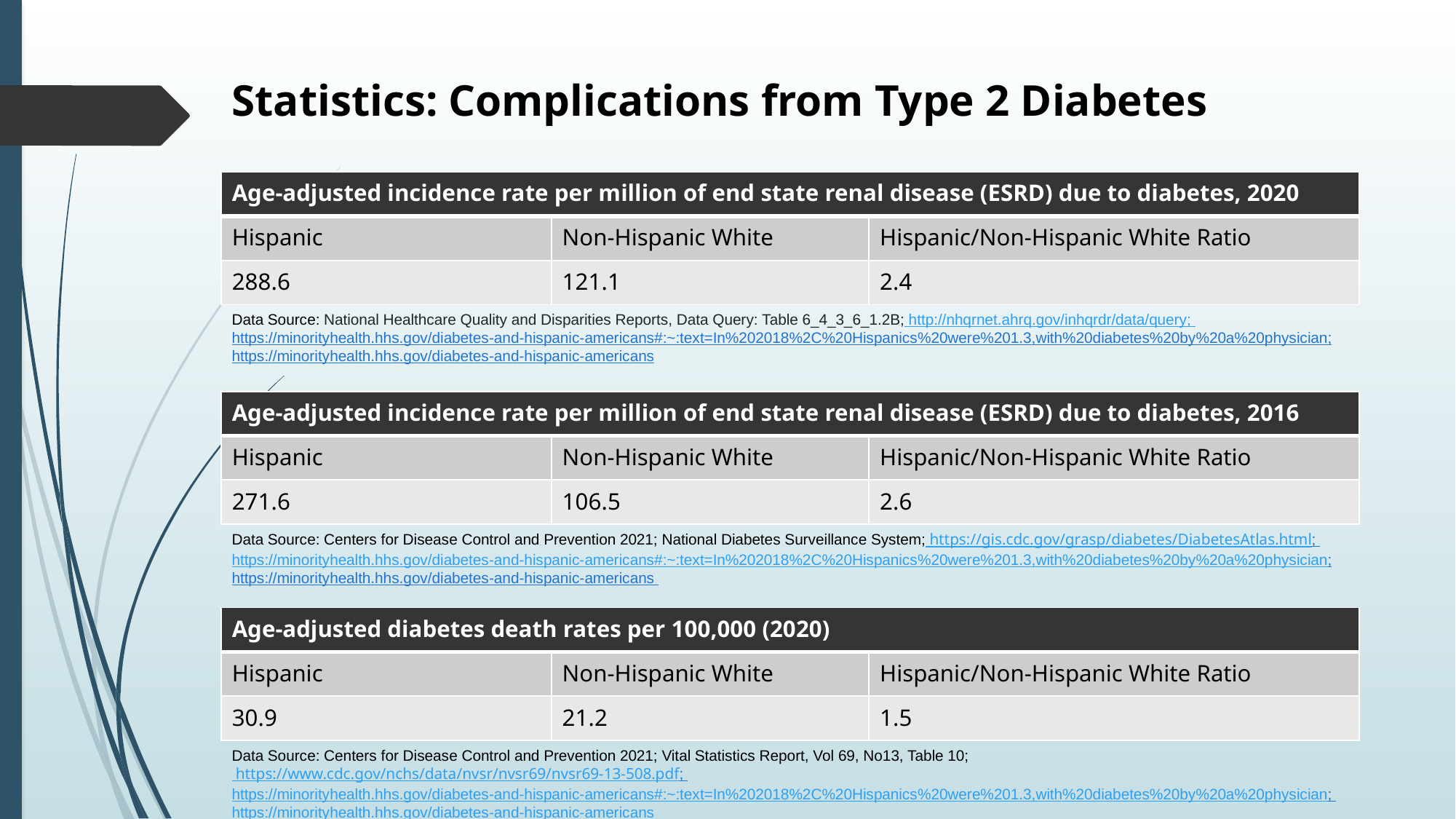

# Statistics: Complications from Type 2 Diabetes
| Age-adjusted incidence rate per million of end state renal disease (ESRD) due to diabetes, 2020 | | |
| --- | --- | --- |
| Hispanic | Non-Hispanic White | Hispanic/Non-Hispanic White Ratio |
| 288.6 | 121.1 | 2.4 |
Data Source: National Healthcare Quality and Disparities Reports, Data Query: Table 6_4_3_6_1.2B; http://nhqrnet.ahrq.gov/inhqrdr/data/query; https://minorityhealth.hhs.gov/diabetes-and-hispanic-americans#:~:text=In%202018%2C%20Hispanics%20were%201.3,with%20diabetes%20by%20a%20physician; https://minorityhealth.hhs.gov/diabetes-and-hispanic-americans
| Age-adjusted incidence rate per million of end state renal disease (ESRD) due to diabetes, 2016 | | |
| --- | --- | --- |
| Hispanic | Non-Hispanic White | Hispanic/Non-Hispanic White Ratio |
| 271.6 | 106.5 | 2.6 |
Data Source: Centers for Disease Control and Prevention 2021; National Diabetes Surveillance System; https://gis.cdc.gov/grasp/diabetes/DiabetesAtlas.html; https://minorityhealth.hhs.gov/diabetes-and-hispanic-americans#:~:text=In%202018%2C%20Hispanics%20were%201.3,with%20diabetes%20by%20a%20physician; https://minorityhealth.hhs.gov/diabetes-and-hispanic-americans
| Age-adjusted diabetes death rates per 100,000 (2020) | | |
| --- | --- | --- |
| Hispanic | Non-Hispanic White | Hispanic/Non-Hispanic White Ratio |
| 30.9 | 21.2 | 1.5 |
Data Source: Centers for Disease Control and Prevention 2021; Vital Statistics Report, Vol 69, No13, Table 10; https://www.cdc.gov/nchs/data/nvsr/nvsr69/nvsr69-13-508.pdf; https://minorityhealth.hhs.gov/diabetes-and-hispanic-americans#:~:text=In%202018%2C%20Hispanics%20were%201.3,with%20diabetes%20by%20a%20physician; https://minorityhealth.hhs.gov/diabetes-and-hispanic-americans

## Slide 10
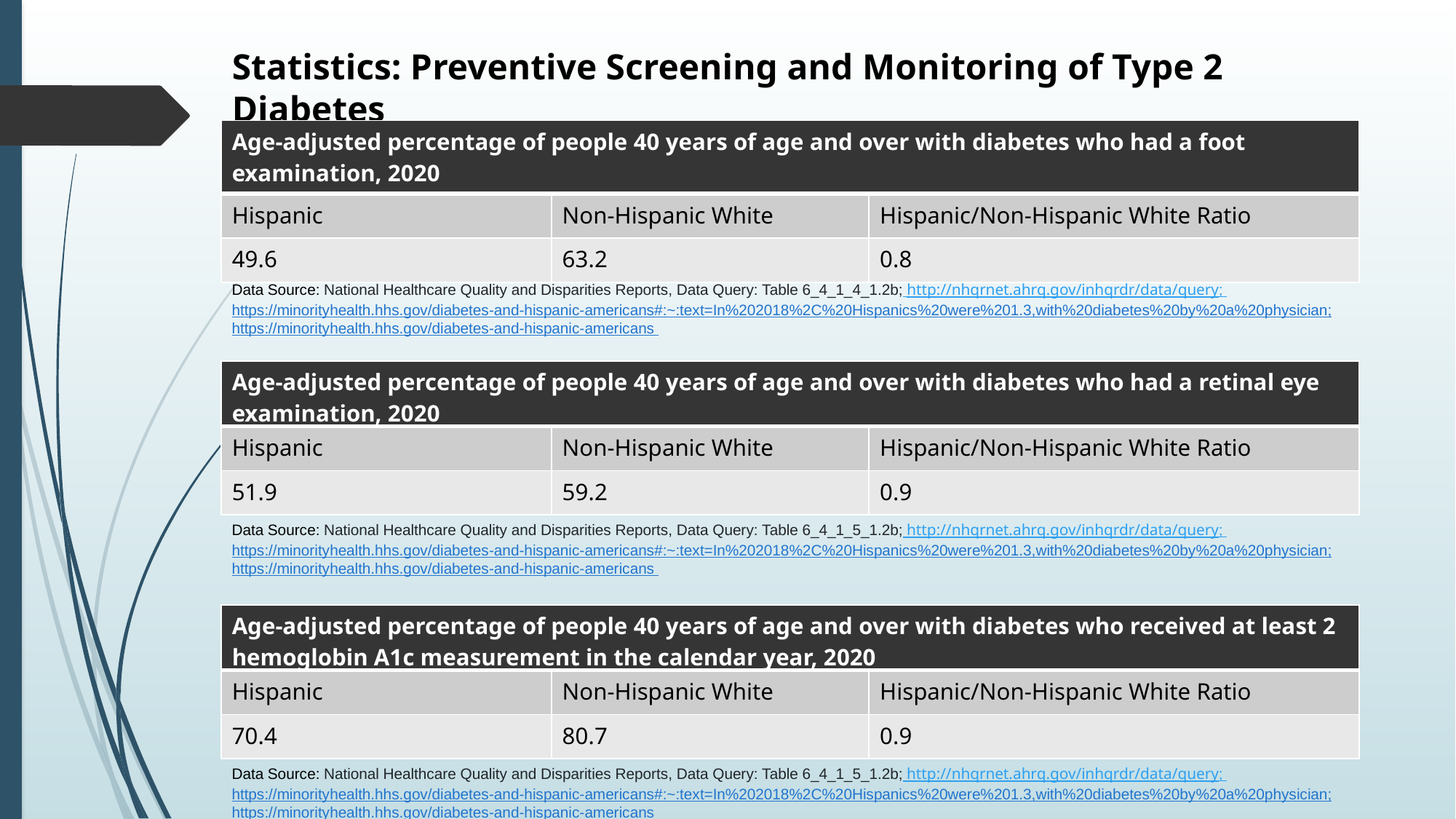

# Statistics: Preventive Screening and Monitoring of Type 2 Diabetes
| Age-adjusted percentage of people 40 years of age and over with diabetes who had a foot examination, 2020 | | |
| --- | --- | --- |
| Hispanic | Non-Hispanic White | Hispanic/Non-Hispanic White Ratio |
| 49.6 | 63.2 | 0.8 |
Data Source: National Healthcare Quality and Disparities Reports, Data Query: Table 6_4_1_4_1.2b; http://nhqrnet.ahrq.gov/inhqrdr/data/query; https://minorityhealth.hhs.gov/diabetes-and-hispanic-americans#:~:text=In%202018%2C%20Hispanics%20were%201.3,with%20diabetes%20by%20a%20physician; https://minorityhealth.hhs.gov/diabetes-and-hispanic-americans
| Age-adjusted percentage of people 40 years of age and over with diabetes who had a retinal eye examination, 2020 | | |
| --- | --- | --- |
| Hispanic | Non-Hispanic White | Hispanic/Non-Hispanic White Ratio |
| 51.9 | 59.2 | 0.9 |
Data Source: National Healthcare Quality and Disparities Reports, Data Query: Table 6_4_1_5_1.2b; http://nhqrnet.ahrq.gov/inhqrdr/data/query; https://minorityhealth.hhs.gov/diabetes-and-hispanic-americans#:~:text=In%202018%2C%20Hispanics%20were%201.3,with%20diabetes%20by%20a%20physician; https://minorityhealth.hhs.gov/diabetes-and-hispanic-americans
| Age-adjusted percentage of people 40 years of age and over with diabetes who received at least 2 hemoglobin A1c measurement in the calendar year, 2020 | | |
| --- | --- | --- |
| Hispanic | Non-Hispanic White | Hispanic/Non-Hispanic White Ratio |
| 70.4 | 80.7 | 0.9 |
Data Source: National Healthcare Quality and Disparities Reports, Data Query: Table 6_4_1_5_1.2b; http://nhqrnet.ahrq.gov/inhqrdr/data/query; https://minorityhealth.hhs.gov/diabetes-and-hispanic-americans#:~:text=In%202018%2C%20Hispanics%20were%201.3,with%20diabetes%20by%20a%20physician; https://minorityhealth.hhs.gov/diabetes-and-hispanic-americans

## Slide 11
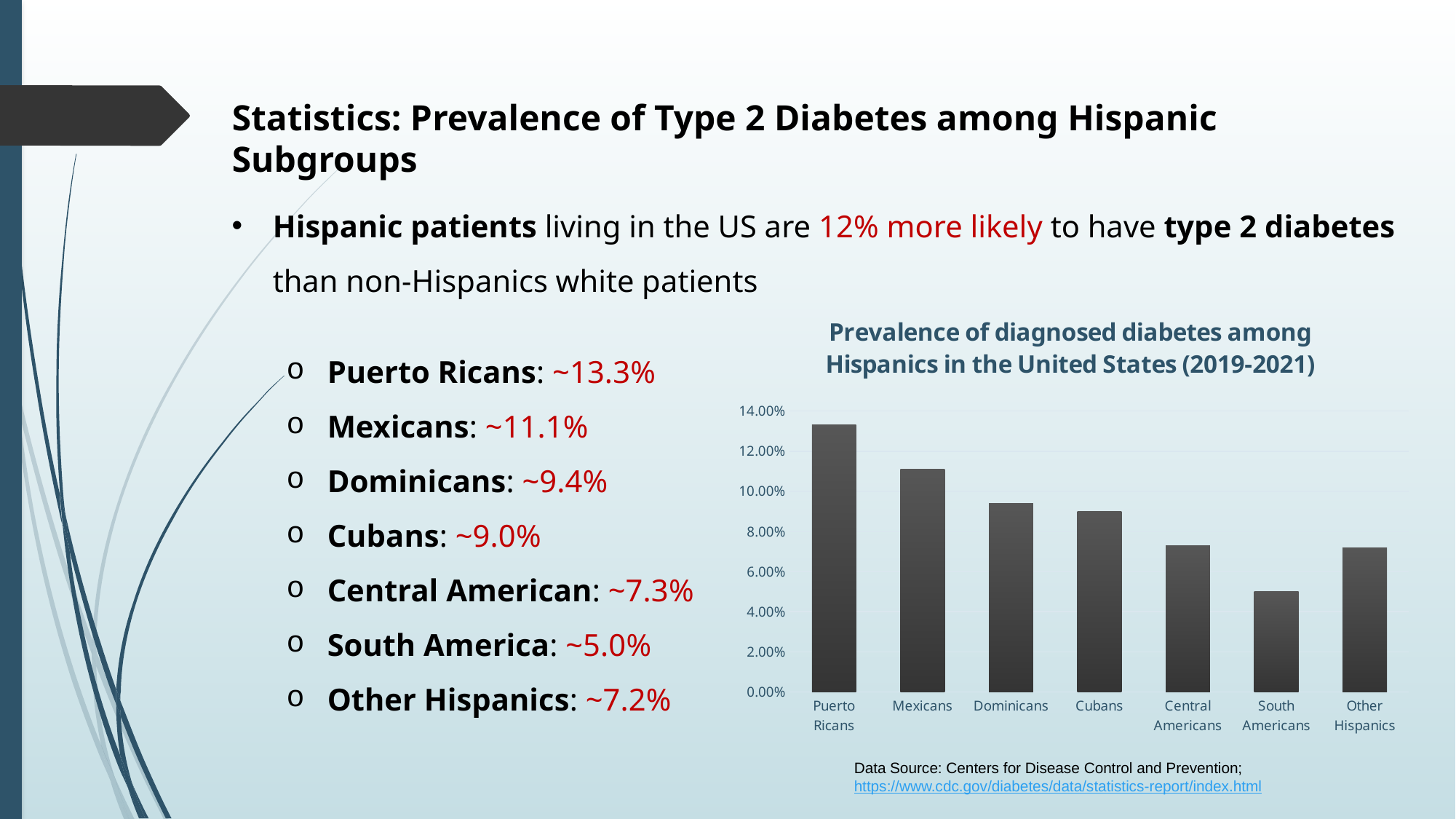

# Statistics: Prevalence of Type 2 Diabetes among Hispanic Subgroups
Hispanic patients living in the US are 12% more likely to have type 2 diabetes than non-Hispanics white patients
Puerto Ricans: ~13.3%
Mexicans: ~11.1%
Dominicans: ~9.4%
Cubans: ~9.0%
Central American: ~7.3%
South America: ~5.0%
Other Hispanics: ~7.2%
### Chart: Prevalence of diagnosed diabetes among Hispanics in the United States (2019-2021)
| Category | |
|---|---|
| Puerto Ricans | 0.133 |
| Mexicans | 0.111 |
| Dominicans | 0.094 |
| Cubans | 0.09 |
| Central Americans | 0.073 |
| South Americans | 0.05 |
| Other Hispanics | 0.072 |Data Source: Centers for Disease Control and Prevention; https://www.cdc.gov/diabetes/data/statistics-report/index.html

## Slide 12
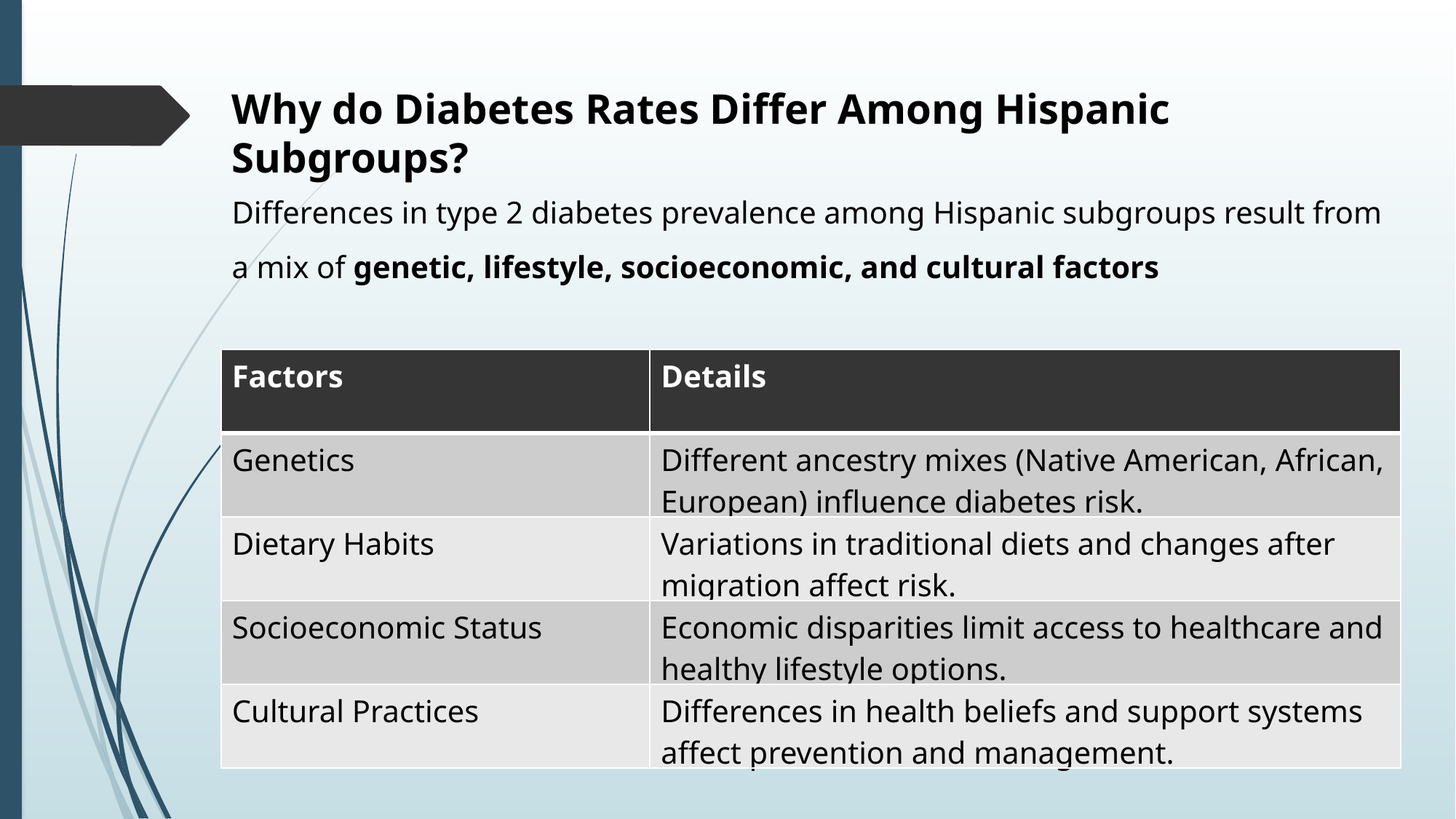

# Why do Diabetes Rates Differ Among Hispanic Subgroups?
Differences in type 2 diabetes prevalence among Hispanic subgroups result from a mix of genetic, lifestyle, socioeconomic, and cultural factors
| Factors | Details |
| --- | --- |
| Genetics | Different ancestry mixes (Native American, African, European) influence diabetes risk. |
| Dietary Habits | Variations in traditional diets and changes after migration affect risk. |
| Socioeconomic Status | Economic disparities limit access to healthcare and healthy lifestyle options. |
| Cultural Practices | Differences in health beliefs and support systems affect prevention and management. |

## Slide 13
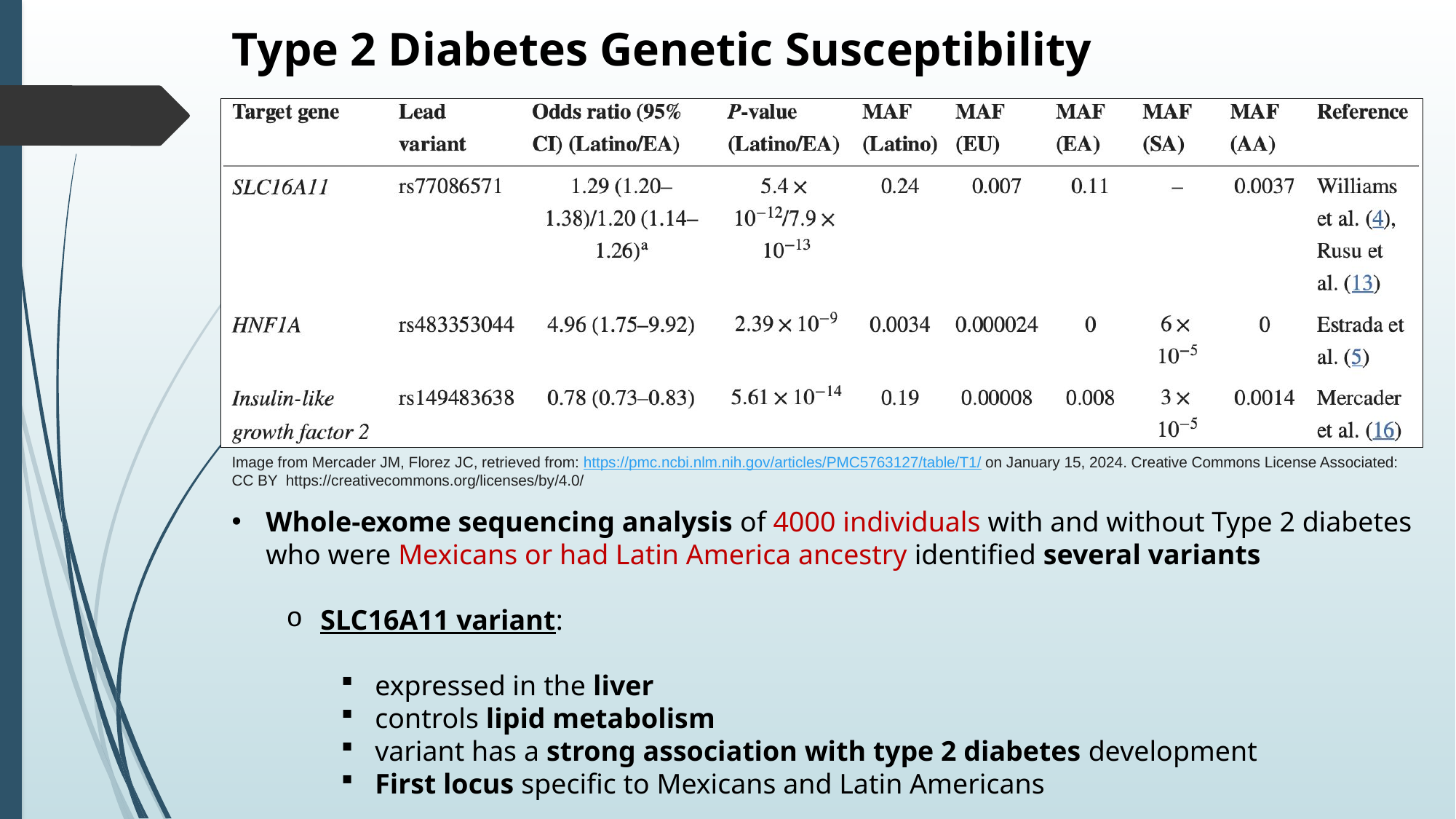

# Type 2 Diabetes Genetic Susceptibility
Image from Mercader JM, Florez JC, retrieved from: https://pmc.ncbi.nlm.nih.gov/articles/PMC5763127/table/T1/ on January 15, 2024. Creative Commons License Associated: CC BY https://creativecommons.org/licenses/by/4.0/
Whole-exome sequencing analysis of 4000 individuals with and without Type 2 diabetes who were Mexicans or had Latin America ancestry identified several variants
SLC16A11 variant:
expressed in the liver
controls lipid metabolism
variant has a strong association with type 2 diabetes development
First locus specific to Mexicans and Latin Americans

## Slide 14
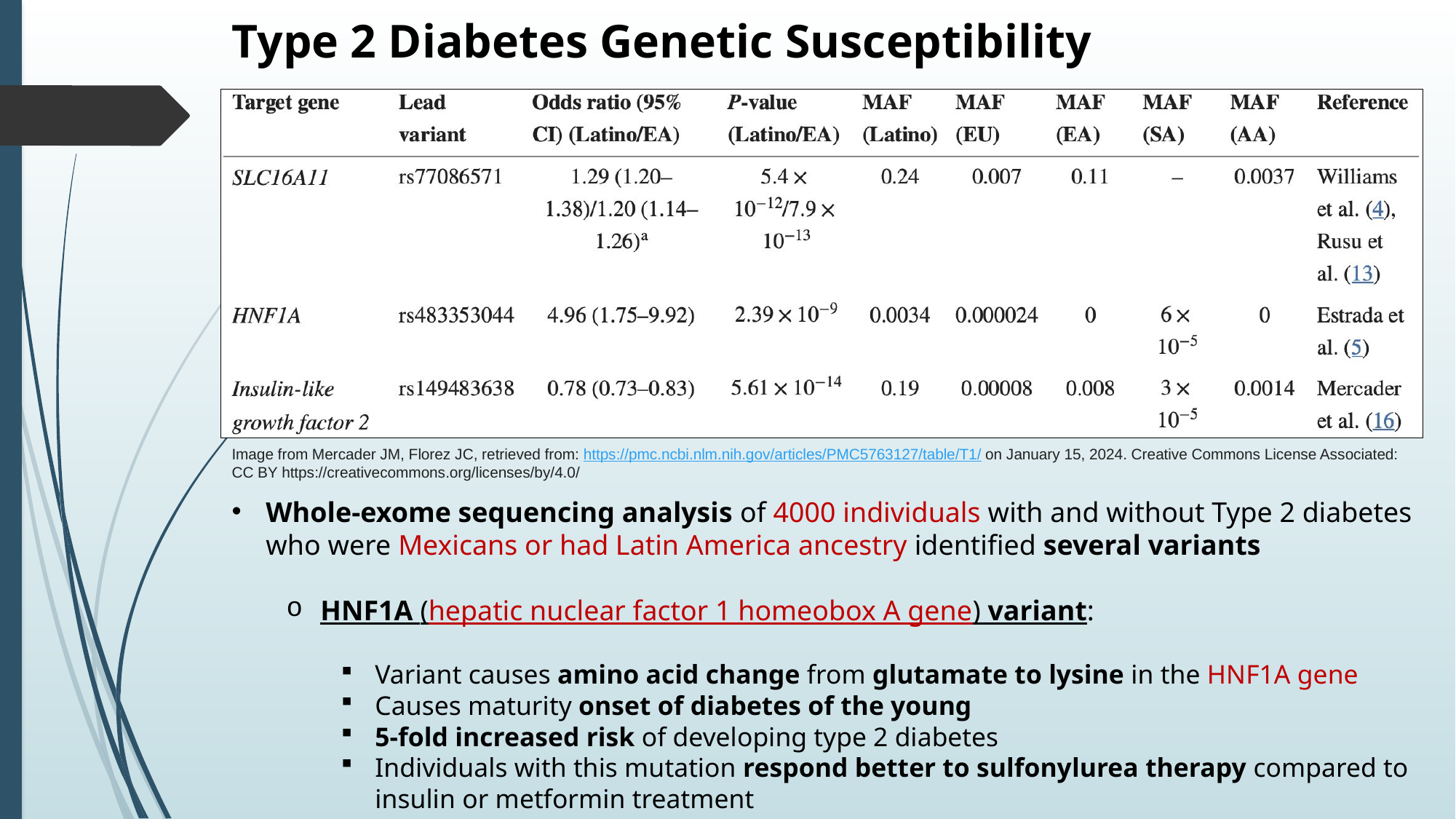

# Type 2 Diabetes Genetic Susceptibility
Image from Mercader JM, Florez JC, retrieved from: https://pmc.ncbi.nlm.nih.gov/articles/PMC5763127/table/T1/ on January 15, 2024. Creative Commons License Associated: CC BY https://creativecommons.org/licenses/by/4.0/
Whole-exome sequencing analysis of 4000 individuals with and without Type 2 diabetes who were Mexicans or had Latin America ancestry identified several variants
HNF1A (hepatic nuclear factor 1 homeobox A gene) variant:
Variant causes amino acid change from glutamate to lysine in the HNF1A gene
Causes maturity onset of diabetes of the young
5-fold increased risk of developing type 2 diabetes
Individuals with this mutation respond better to sulfonylurea therapy compared to insulin or metformin treatment

## Slide 15
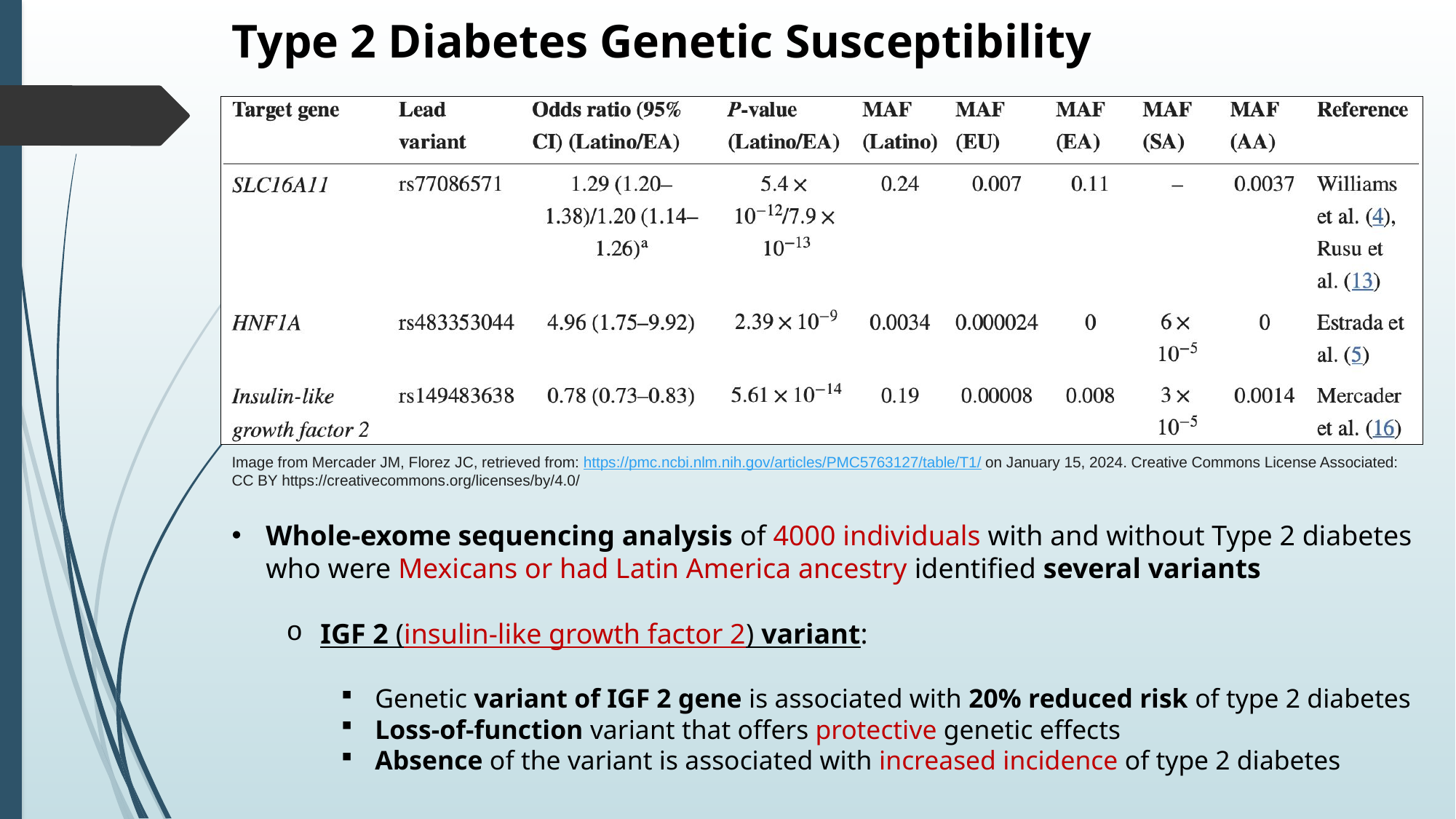

# Type 2 Diabetes Genetic Susceptibility
Image from Mercader JM, Florez JC, retrieved from: https://pmc.ncbi.nlm.nih.gov/articles/PMC5763127/table/T1/ on January 15, 2024. Creative Commons License Associated: CC BY https://creativecommons.org/licenses/by/4.0/
Whole-exome sequencing analysis of 4000 individuals with and without Type 2 diabetes who were Mexicans or had Latin America ancestry identified several variants
IGF 2 (insulin-like growth factor 2) variant:
Genetic variant of IGF 2 gene is associated with 20% reduced risk of type 2 diabetes
Loss-of-function variant that offers protective genetic effects
Absence of the variant is associated with increased incidence of type 2 diabetes

## Slide 16
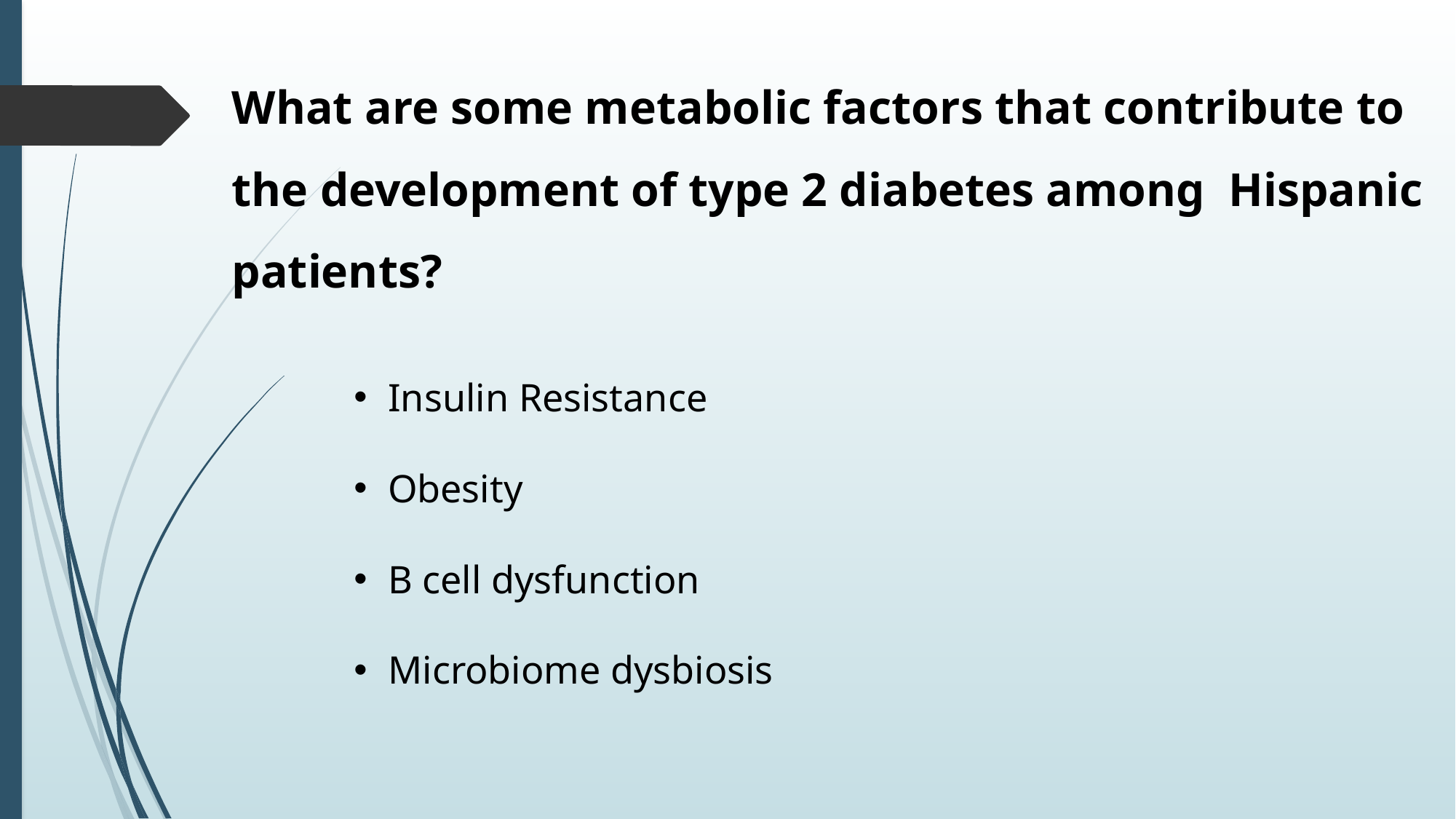

# What are some metabolic factors that contribute to the development of type 2 diabetes among Hispanic patients?
Insulin Resistance
Obesity
Β cell dysfunction
Microbiome dysbiosis

## Slide 17
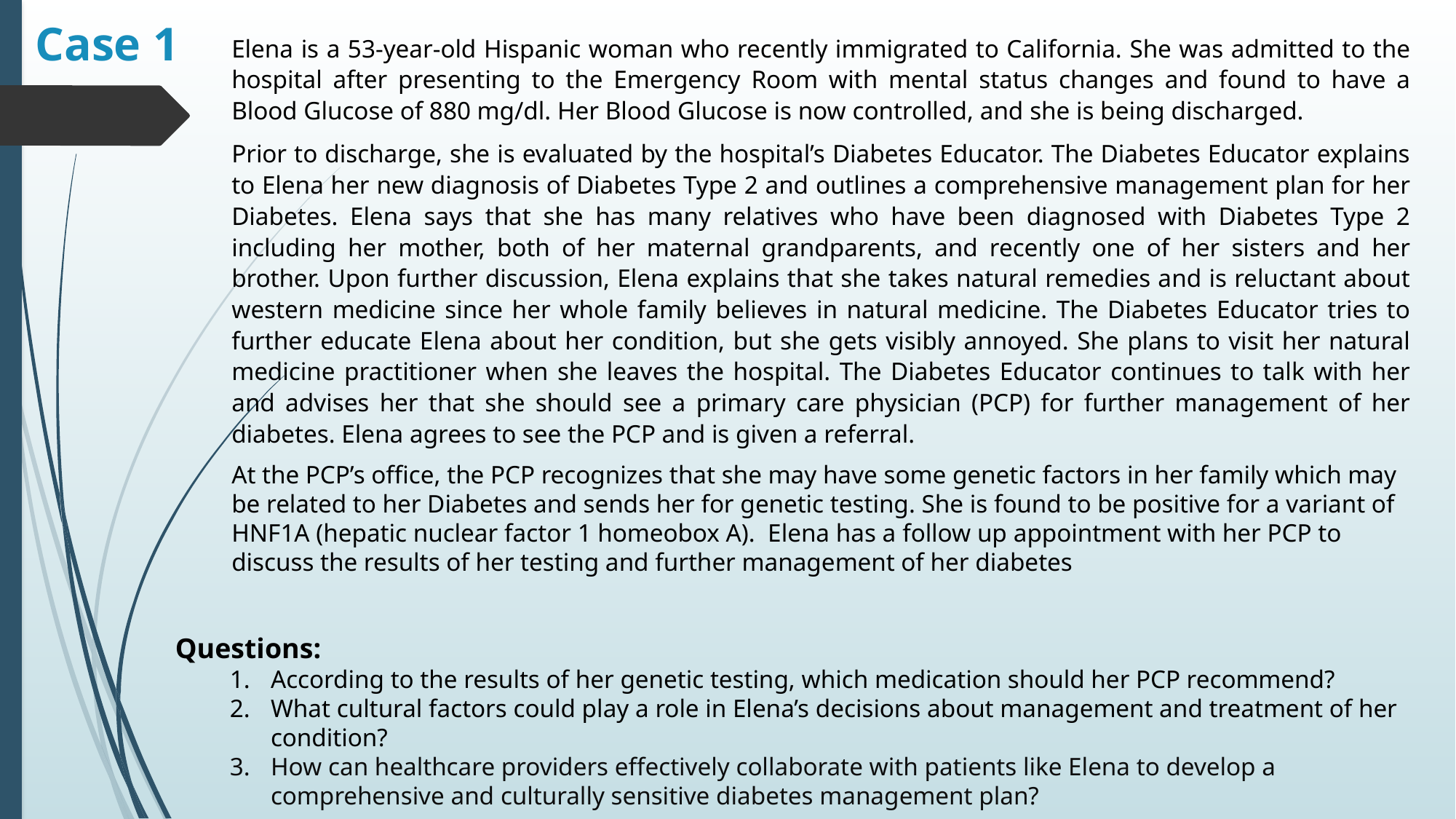

# Case 1
Elena is a 53-year-old Hispanic woman who recently immigrated to California. She was admitted to the hospital after presenting to the Emergency Room with mental status changes and found to have a Blood Glucose of 880 mg/dl. Her Blood Glucose is now controlled, and she is being discharged.
Prior to discharge, she is evaluated by the hospital’s Diabetes Educator. The Diabetes Educator explains to Elena her new diagnosis of Diabetes Type 2 and outlines a comprehensive management plan for her Diabetes. Elena says that she has many relatives who have been diagnosed with Diabetes Type 2 including her mother, both of her maternal grandparents, and recently one of her sisters and her brother. Upon further discussion, Elena explains that she takes natural remedies and is reluctant about western medicine since her whole family believes in natural medicine. The Diabetes Educator tries to further educate Elena about her condition, but she gets visibly annoyed. She plans to visit her natural medicine practitioner when she leaves the hospital. The Diabetes Educator continues to talk with her and advises her that she should see a primary care physician (PCP) for further management of her diabetes. Elena agrees to see the PCP and is given a referral.
At the PCP’s office, the PCP recognizes that she may have some genetic factors in her family which may be related to her Diabetes and sends her for genetic testing. She is found to be positive for a variant of HNF1A (hepatic nuclear factor 1 homeobox A). Elena has a follow up appointment with her PCP to discuss the results of her testing and further management of her diabetes
Questions:
According to the results of her genetic testing, which medication should her PCP recommend?
What cultural factors could play a role in Elena’s decisions about management and treatment of her condition?
How can healthcare providers effectively collaborate with patients like Elena to develop a comprehensive and culturally sensitive diabetes management plan?

## Slide 18
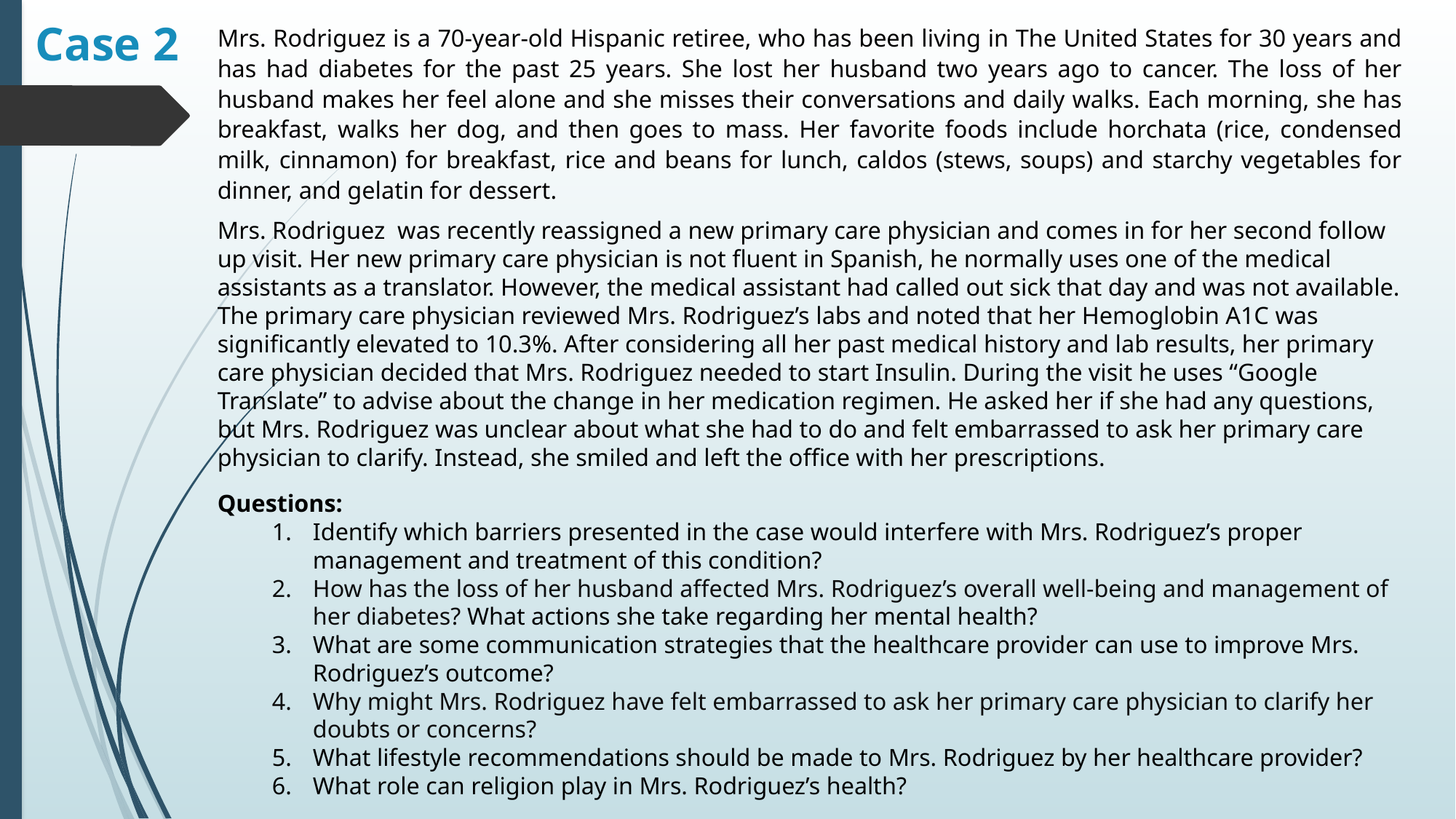

# Case 2
Mrs. Rodriguez is a 70-year-old Hispanic retiree, who has been living in The United States for 30 years and has had diabetes for the past 25 years. She lost her husband two years ago to cancer. The loss of her husband makes her feel alone and she misses their conversations and daily walks. Each morning, she has breakfast, walks her dog, and then goes to mass. Her favorite foods include horchata (rice, condensed milk, cinnamon) for breakfast, rice and beans for lunch, caldos (stews, soups) and starchy vegetables for dinner, and gelatin for dessert.
Mrs. Rodriguez was recently reassigned a new primary care physician and comes in for her second follow up visit. Her new primary care physician is not fluent in Spanish, he normally uses one of the medical assistants as a translator. However, the medical assistant had called out sick that day and was not available. The primary care physician reviewed Mrs. Rodriguez’s labs and noted that her Hemoglobin A1C was significantly elevated to 10.3%. After considering all her past medical history and lab results, her primary care physician decided that Mrs. Rodriguez needed to start Insulin. During the visit he uses “Google Translate” to advise about the change in her medication regimen. He asked her if she had any questions, but Mrs. Rodriguez was unclear about what she had to do and felt embarrassed to ask her primary care physician to clarify. Instead, she smiled and left the office with her prescriptions.
Questions:
Identify which barriers presented in the case would interfere with Mrs. Rodriguez’s proper management and treatment of this condition?
How has the loss of her husband affected Mrs. Rodriguez’s overall well-being and management of her diabetes? What actions she take regarding her mental health?
What are some communication strategies that the healthcare provider can use to improve Mrs. Rodriguez’s outcome?
Why might Mrs. Rodriguez have felt embarrassed to ask her primary care physician to clarify her doubts or concerns?
What lifestyle recommendations should be made to Mrs. Rodriguez by her healthcare provider?
What role can religion play in Mrs. Rodriguez’s health?

## Slide 19
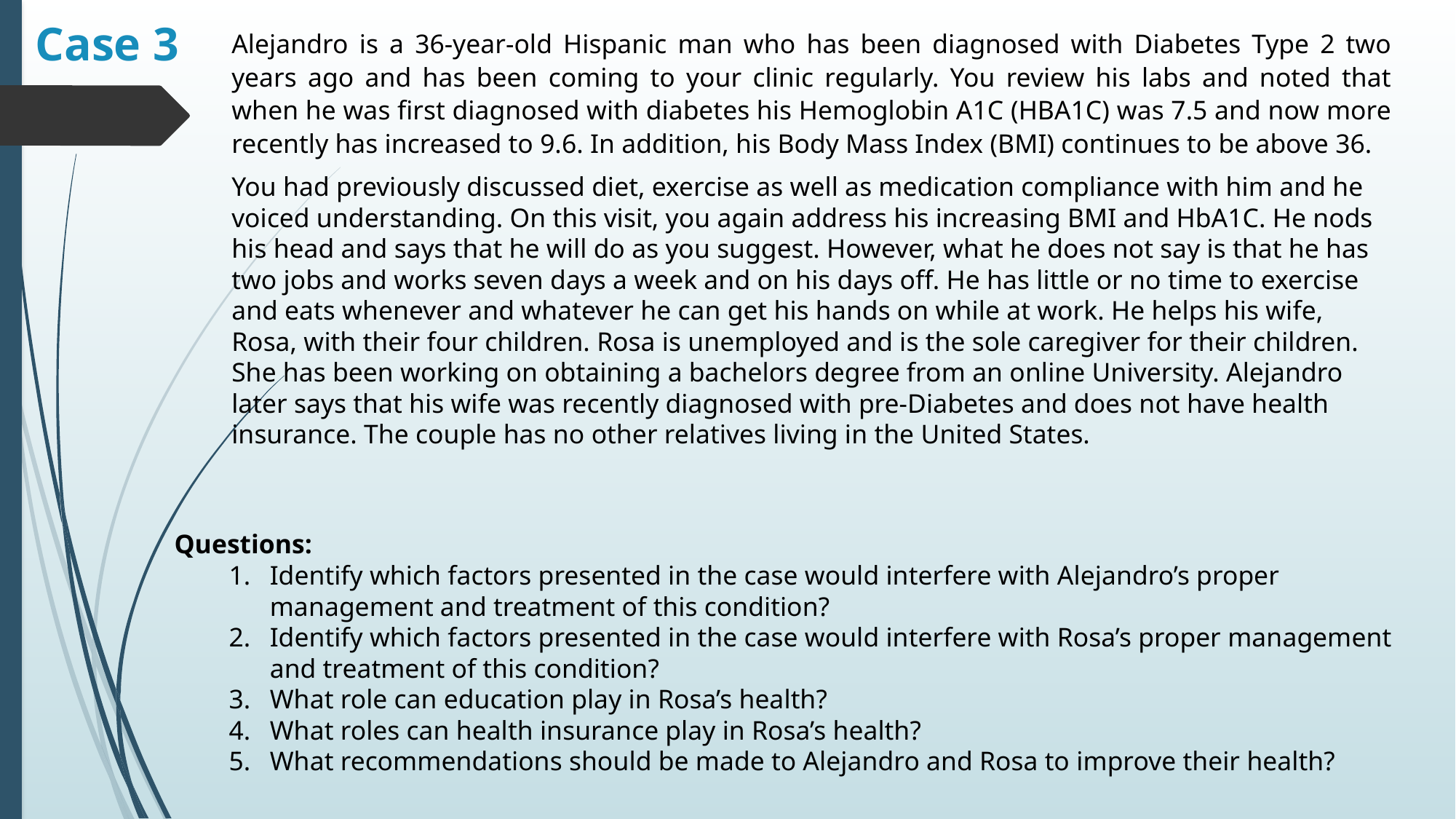

# Case 3
Alejandro is a 36-year-old Hispanic man who has been diagnosed with Diabetes Type 2 two years ago and has been coming to your clinic regularly. You review his labs and noted that when he was first diagnosed with diabetes his Hemoglobin A1C (HBA1C) was 7.5 and now more recently has increased to 9.6. In addition, his Body Mass Index (BMI) continues to be above 36.
You had previously discussed diet, exercise as well as medication compliance with him and he voiced understanding. On this visit, you again address his increasing BMI and HbA1C. He nods his head and says that he will do as you suggest. However, what he does not say is that he has two jobs and works seven days a week and on his days off. He has little or no time to exercise and eats whenever and whatever he can get his hands on while at work. He helps his wife, Rosa, with their four children. Rosa is unemployed and is the sole caregiver for their children. She has been working on obtaining a bachelors degree from an online University. Alejandro later says that his wife was recently diagnosed with pre-Diabetes and does not have health insurance. The couple has no other relatives living in the United States.
Questions:
Identify which factors presented in the case would interfere with Alejandro’s proper management and treatment of this condition?
Identify which factors presented in the case would interfere with Rosa’s proper management and treatment of this condition?
What role can education play in Rosa’s health?
What roles can health insurance play in Rosa’s health?
What recommendations should be made to Alejandro and Rosa to improve their health?

## Slide 20
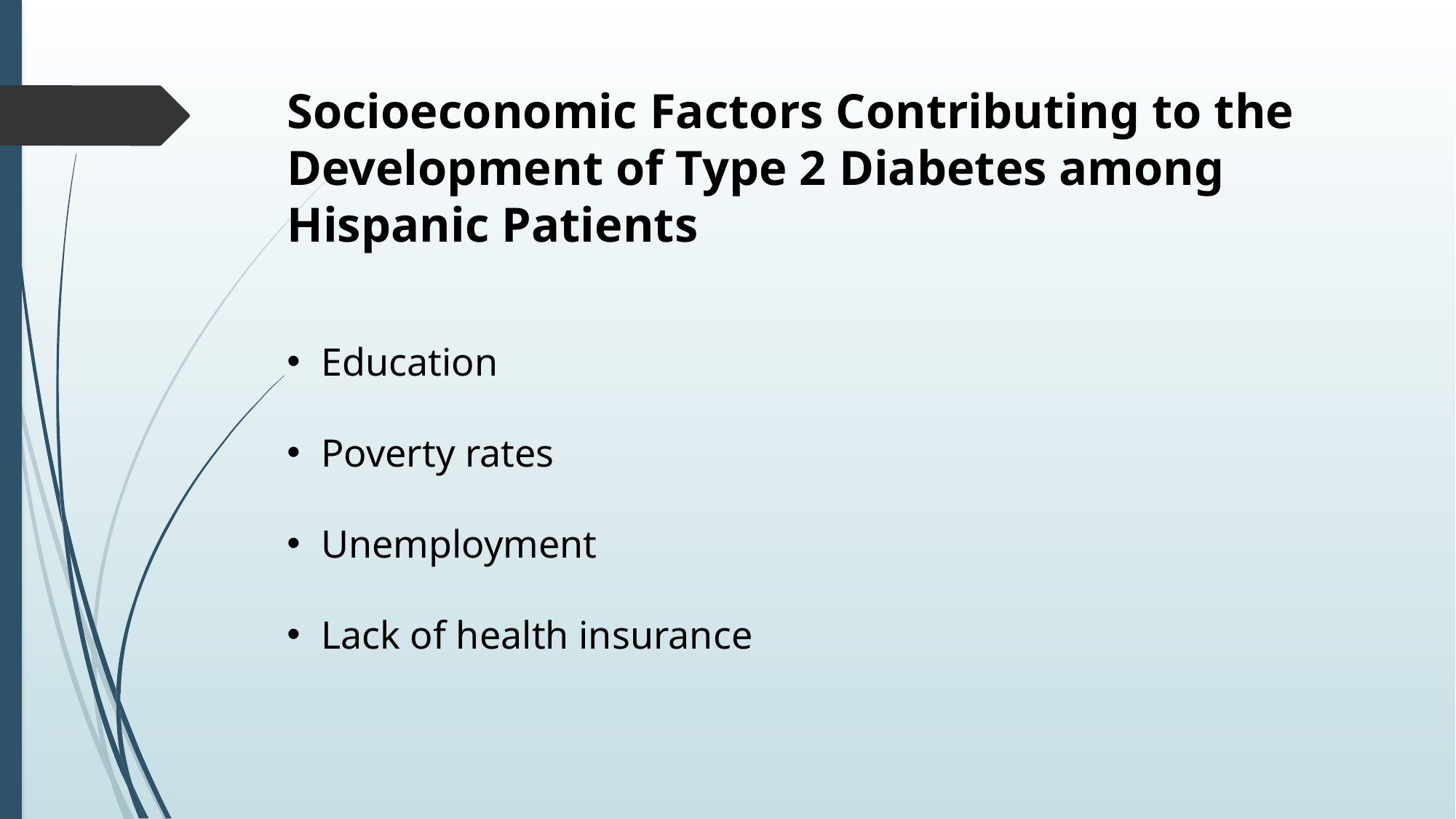

# Socioeconomic Factors Contributing to the Development of Type 2 Diabetes among Hispanic Patients
Education
Poverty rates
Unemployment
Lack of health insurance

## Slide 21
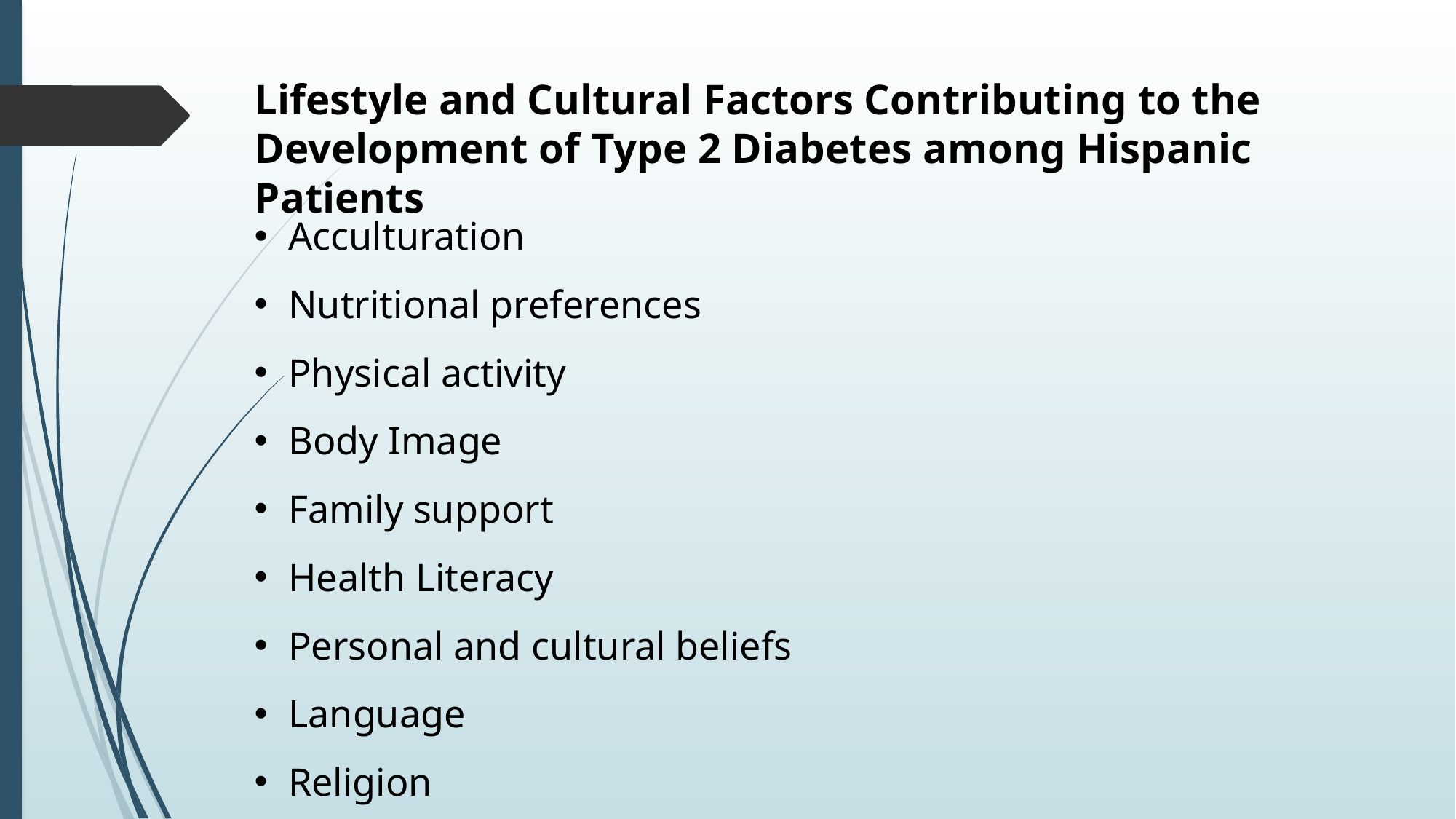

# Lifestyle and Cultural Factors Contributing to the Development of Type 2 Diabetes among Hispanic Patients
Acculturation
Nutritional preferences
Physical activity
Body Image
Family support
Health Literacy
Personal and cultural beliefs
Language
Religion

## Slide 22
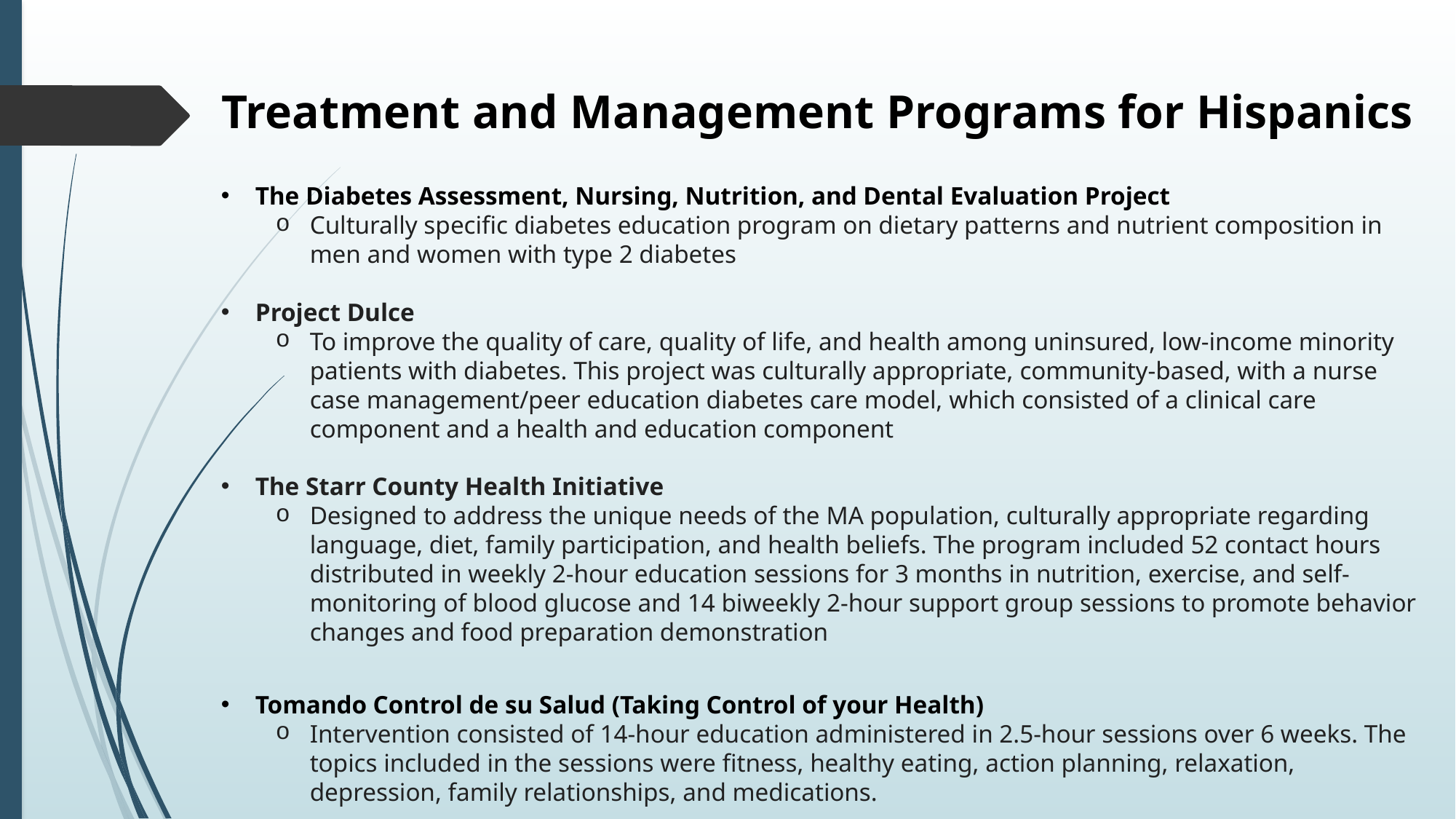

# Treatment and Management Programs for Hispanics
The Diabetes Assessment, Nursing, Nutrition, and Dental Evaluation Project
Culturally specific diabetes education program on dietary patterns and nutrient composition in men and women with type 2 diabetes
Project Dulce
To improve the quality of care, quality of life, and health among uninsured, low-income minority patients with diabetes. This project was culturally appropriate, community-based, with a nurse case management/peer education diabetes care model, which consisted of a clinical care component and a health and education component
The Starr County Health Initiative
Designed to address the unique needs of the MA population, culturally appropriate regarding language, diet, family participation, and health beliefs. The program included 52 contact hours distributed in weekly 2-hour education sessions for 3 months in nutrition, exercise, and self-monitoring of blood glucose and 14 biweekly 2-hour support group sessions to promote behavior changes and food preparation demonstration
Tomando Control de su Salud (Taking Control of your Health)
Intervention consisted of 14-hour education administered in 2.5-hour sessions over 6 weeks. The topics included in the sessions were fitness, healthy eating, action planning, relaxation, depression, family relationships, and medications.

## Slide 23
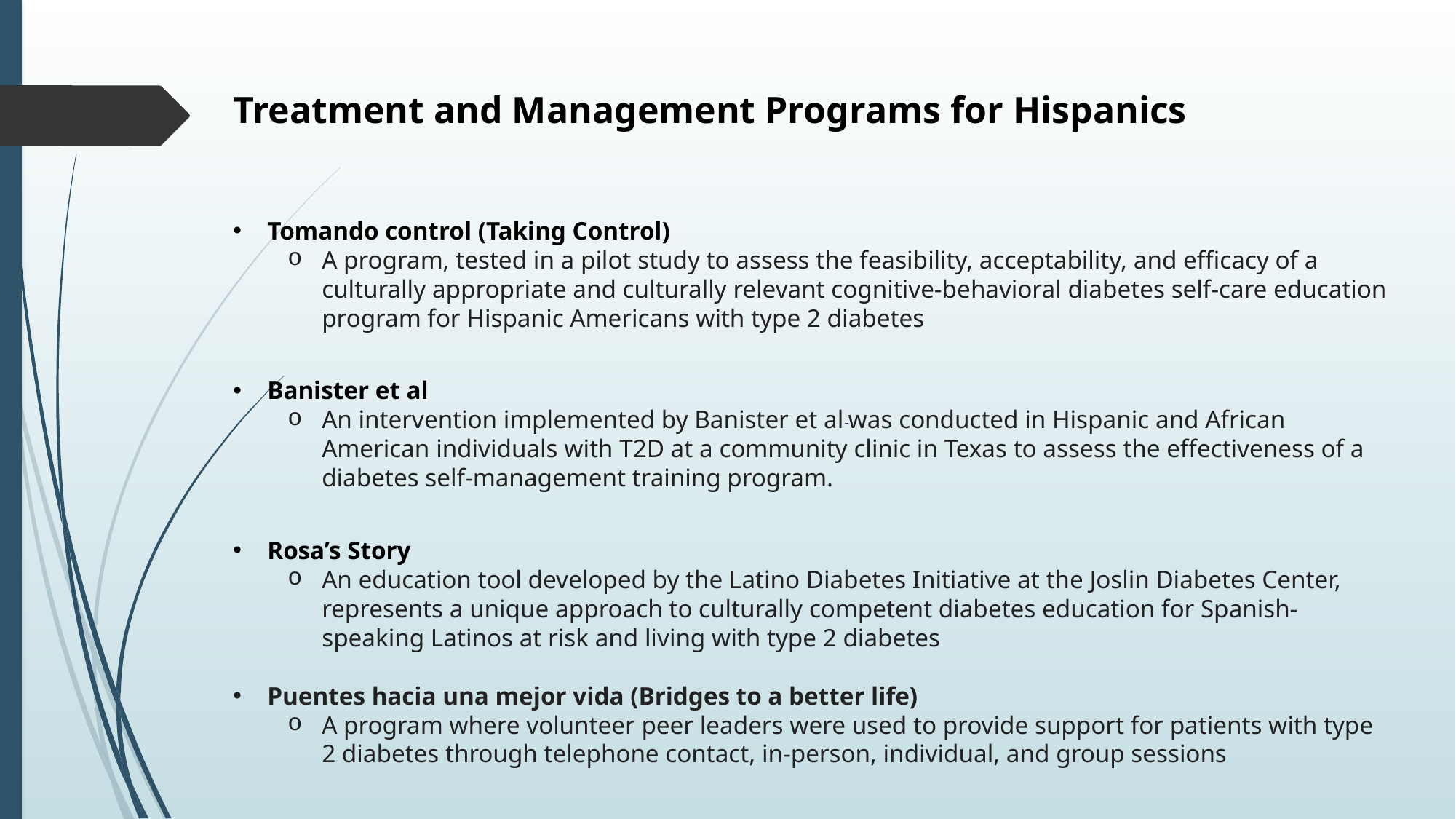

# Treatment and Management Programs for Hispanics
Tomando control (Taking Control)
A program, tested in a pilot study to assess the feasibility, acceptability, and efficacy of a culturally appropriate and culturally relevant cognitive-behavioral diabetes self-care education program for Hispanic Americans with type 2 diabetes
Banister et al
An intervention implemented by Banister et al was conducted in Hispanic and African American individuals with T2D at a community clinic in Texas to assess the effectiveness of a diabetes self-management training program.
Rosa’s Story
An education tool developed by the Latino Diabetes Initiative at the Joslin Diabetes Center, represents a unique approach to culturally competent diabetes education for Spanish-speaking Latinos at risk and living with type 2 diabetes
Puentes hacia una mejor vida (Bridges to a better life)
A program where volunteer peer leaders were used to provide support for patients with type 2 diabetes through telephone contact, in-person, individual, and group sessions

## Slide 24
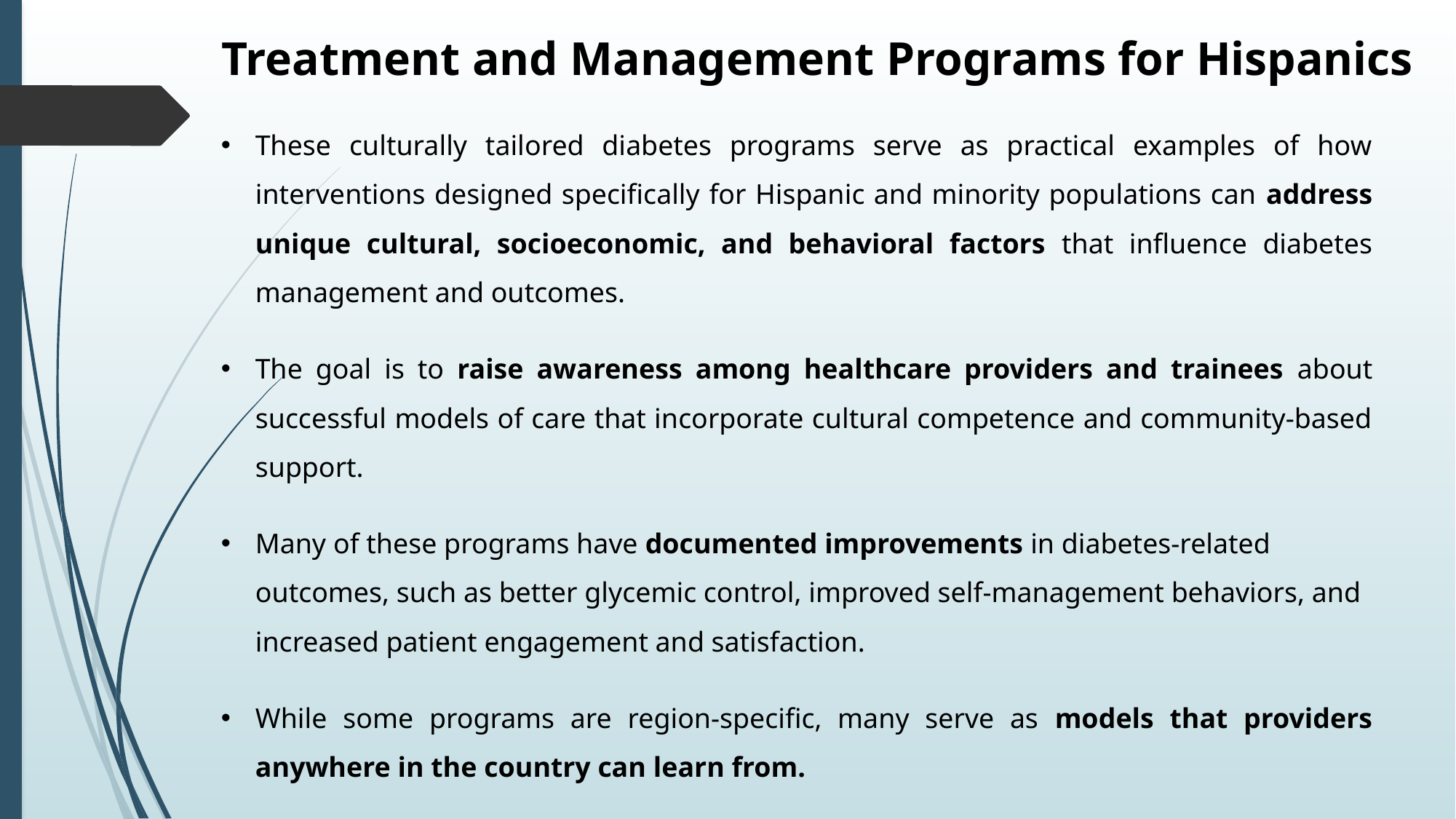

# Treatment and Management Programs for Hispanics
These culturally tailored diabetes programs serve as practical examples of how interventions designed specifically for Hispanic and minority populations can address unique cultural, socioeconomic, and behavioral factors that influence diabetes management and outcomes.
The goal is to raise awareness among healthcare providers and trainees about successful models of care that incorporate cultural competence and community-based support.
Many of these programs have documented improvements in diabetes-related outcomes, such as better glycemic control, improved self-management behaviors, and increased patient engagement and satisfaction.
While some programs are region-specific, many serve as models that providers anywhere in the country can learn from.

## Slide 25
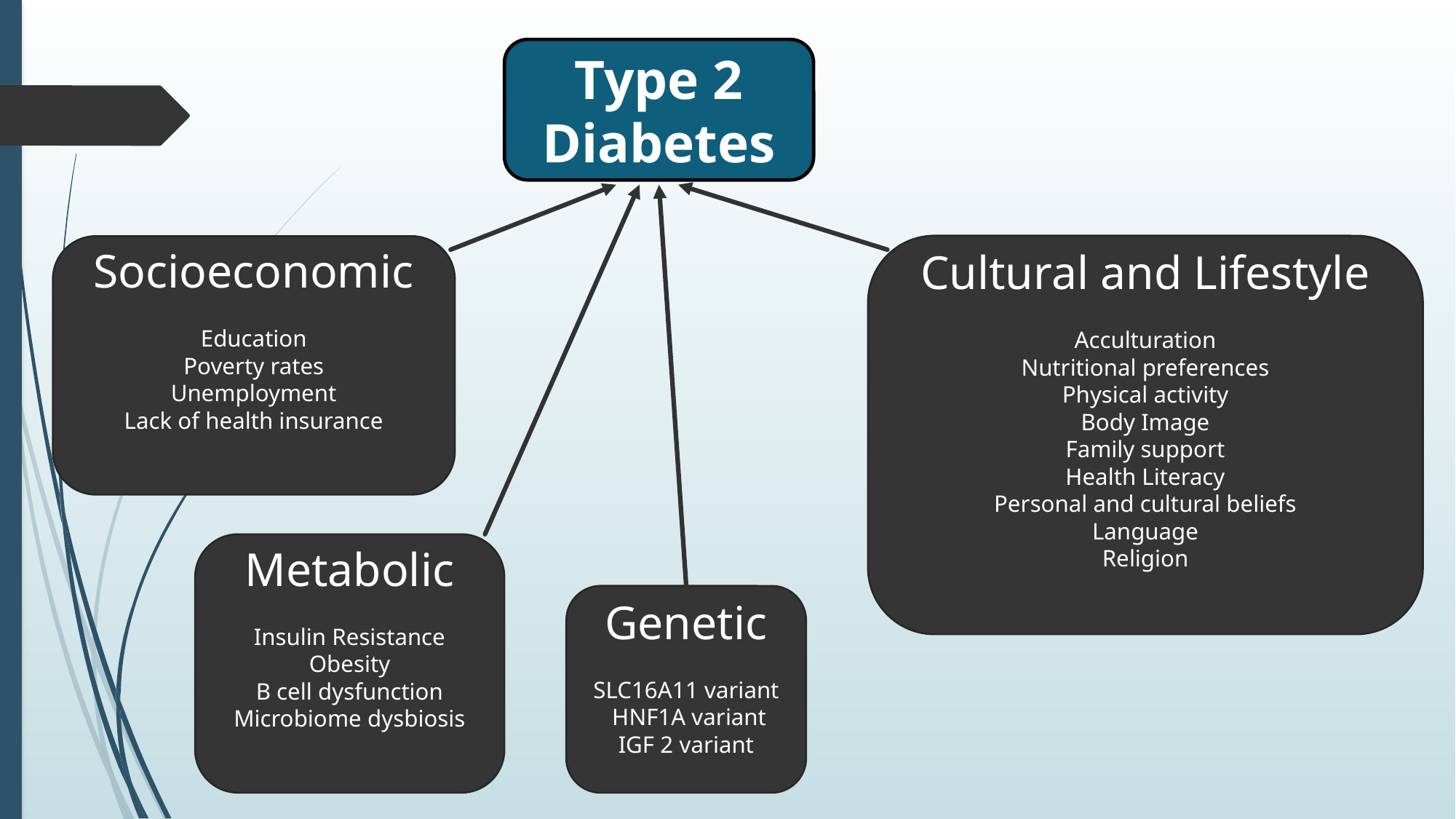

Type 2 Diabetes
Cultural and Lifestyle
Acculturation
Nutritional preferences
Physical activity
Body Image
Family support
Health Literacy
Personal and cultural beliefs
Language
Religion
Socioeconomic
Education
Poverty rates
Unemployment
Lack of health insurance
Metabolic
Insulin Resistance
Obesity
Β cell dysfunction
Microbiome dysbiosis
Genetic
SLC16A11 variant
 HNF1A variant
IGF 2 variant

## Slide 26
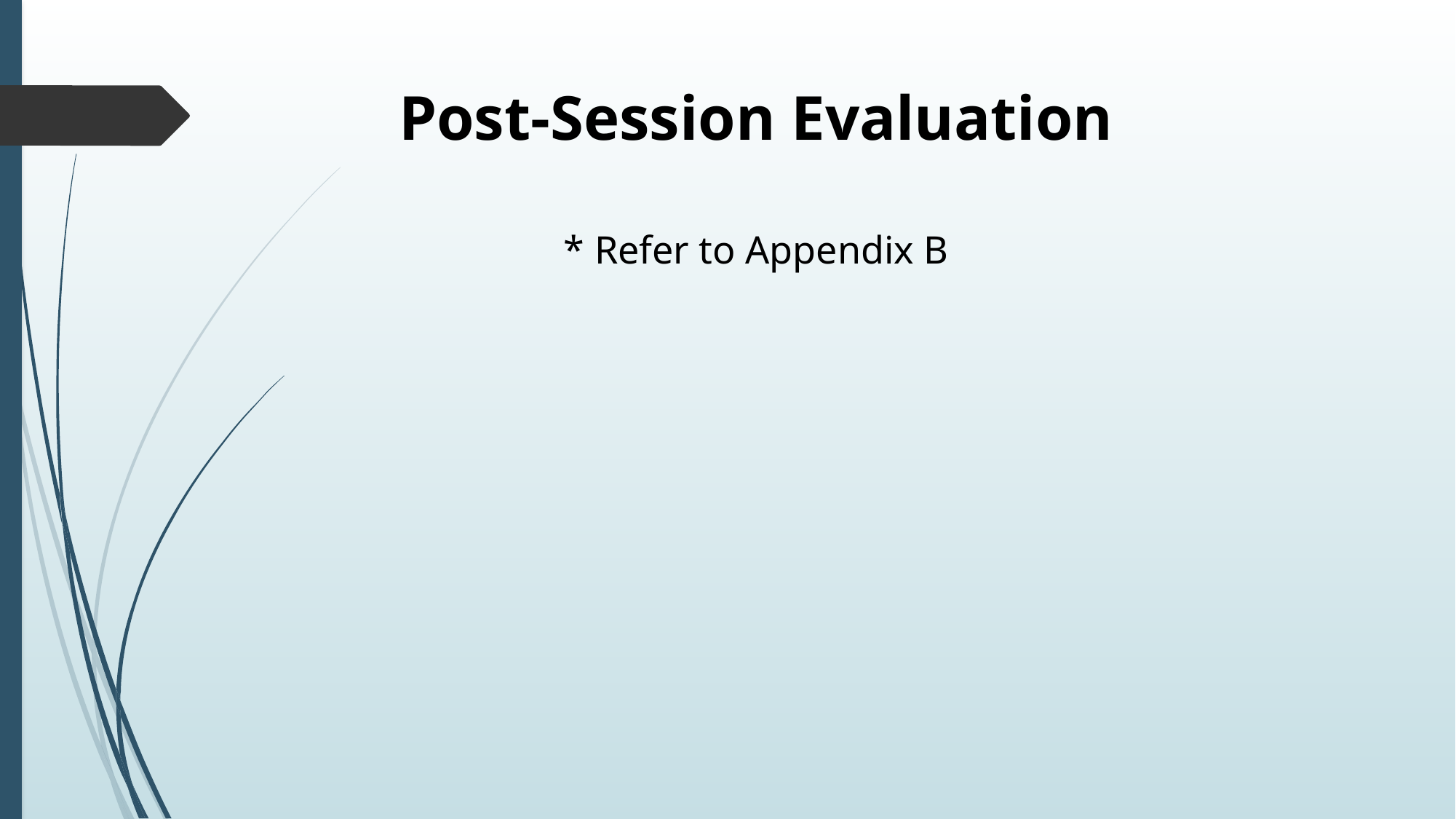

# Post-Session Evaluation
* Refer to Appendix B
