# Supplementary material for: Health Disparities Among Hispanic Patients With Type 2 Diabetes in the United States: An Educational Workshop
Source: MedEdPORTAL. 2026 Jul 22;22:11622. doi: 10.15766/mep_2374-8265.11622 (PMC13388404; doi:10.15766/mep_2374-8265.11622)
Supplement: Supplementary file 1 — Presession Evaluation.docxPostsession Evaluation.docxPresentation.pptxFacilitator Guide.docx [file mep_2374-8265.11622-s001.zip › D. Facilitator Guide.docx]

**Facilitator Guide: Health Disparities among Hispanic Patients with Type 2 Diabetes in the United States: An Educational Workshop**

**Overall Goals**

This workshop aims to help health professionals’ trainees gain awareness of the disparities among Hispanics with type 2 diabetes (T2D) in the United States, while underlying the importance of structural, social, economic, and biological factors that contribute to a greater risk of the disease in this population. Facilitators are encouraged to frame discussions using principles of cultural humility and structural awareness rather than assuming uniform cultural practices across Hispanic populations

**Objectives**

1. Describe health disparities statistics and reports about Hispanic populations with T2D in the United States.
2. Analyze genetic susceptibility and metabolic factors that contribute to T2D among Hispanics
3. Describe cultural, socioeconomic, and lifestyle factors associated with the incidence of T2D among Hispanics
4. Illustrate through cases the need for more for more inclusive and tailored treatment options, preventive education, lifestyle changes, and health interventions that are necessary to improve patient outcome

**Suggested Agenda and Timeline**

- Pre-evaluation (5 minutes)
- PowerPoint Presentation (50 minutes)
- Introduction (12 minutes)
- Health disparities statistics and reports (5 minutes)
- Genetic susceptibility (5 minutes)
- Metabolic Factors (2 minutes)
- Case studies and discussion (25 minutes)
- Read and discuss in a small group (15 minutes)
- Discuss important points in a large group (10 minutes)
- Summary (13 minutes)
- Socioeconomic, lifestyle, and cultural factors (5 minutes)
- Treatment and management programs (5 minutes)
- Integration (3 minutes)
- Post-evaluation (5 minutes)

**Handouts and Materials**

- Computer with connection to projector
- Internet
- Facilitator Instructional Guide
- Pre-evaluation form
- Post-evaluation form
- PowerPoint presentation
- Case studies

**Slide Instructions**

**Slide 1:** Title: **Health Disparities among Hispanics with T2D in The United States**

*The facilitator(s) should introduce themselves to the audience and discuss their role in their respective institution(s)*

**Slide 2:** Pre-Evaluation QR Code

*The facilitator(s) will indicate to the audience to access the pre-evaluation using the QR code and allow time to complete it.*

- QR code must be created for the pre-evaluation

**Slide 3:** Educational Objectives

*The facilitator(s) will present the objectives for the educational intervention.*

**Slide 4:** Agenda

*The facilitator(s) will present the agenda for the educational intervention.*

**Slide 5:** Statistics according to Race and Ethnicity

*The facilitator(s) will give a brief introduction to the importance of understanding health disparities among Hispanics with T2D in the United States. Statistics and reports will be presented to support the educational intervention (references).*

- According to the CDC, T2D accounts for 90 to 95% of all diagnosed cases of diabetes in adults in the U.S.
- T2D affects 11.6% of the U.S. population (38.4 million) with a disproportionate number being of Hispanic descent
- Hispanics have the third-highest prevalence of all ethnicities with T2D in U.S. (11.7%)

**Slide 6:** Statistics

*The facilitator(s) will provide statistics and reports to support the educational intervention.*

- Over their lifetime, U.S. adults have 40% chance of developing T2D
- Hispanic adults have more than a 50% chance of developing T2D
  - More likely to develop it at a younger age (SEARCH multicenter based study: T2D in young individuals was higher in Hispanics (46.1%) than non-Hispanic white (14%)
  - Higher risk of developing complications such as kidney failure and vision loss
- Death rates from T2D in Hispanic populations are higher than those of non-Hispanic white populations.

**Slide 7:** Statistics: Diagnosed Cases of Type 2 Diabetes

*The facilitator will explain the ratios of diagnosed cases of T2D among Hispanics compared to non-Hispanic Whites.*

- According to the CDC, the ratio of Hispanics to Non-Hispanic white adults age 18 and over that have been diagnosed with T2D is about 1.6, with men having a greater rate of incidence compared to women

**Slide 8:** Statistics: Complications from Type 2 Diabetes

*The facilitator will explain the rate of health complications related to T2D among Hispanics compared to non-Hispanic Whites in the United States.*

- According to the CDC, Hispanics have a 1.4 higher rate of visual impairments related to T2D in comparison to non-Hispanic whites
- According to the National Healthcare Quality and Disparities Reports, Hispanics have a 1.6- and 1.7-fold higher rates of hospital admissions and lower extremities amputations respectively due to T2D compared to non-Hispanic whites.

**Slide 9:** Statistics: Complications from Type 2 Diabetes *(cont.)*

*The facilitator will continue explaining the rate of health complications related to T2D among Hispanics compared to non-Hispanic whites in the United States.*

- According to the National Healthcare Quality and Disparities Reports, Hispanics have a 2.4-fold higher rate of developing end state renal disease (ESRD) due to T2D in comparison to non-Hispanic whites
- According to the CDC, Hispanics have 2.6- and 1.5-fold higher rates of ESDR and death related to T2D compared to non-Hispanic whites.

**Slide 10:** Statistics: Preventive Screening and Monitoring of Type 2 Diabetes

*The facilitator will compare the rates of preventive T2D exams performed on Hispanics and non-Hispanic whites.*

- According to the National Healthcare Quality and Disparities Reports, Hispanics have a 0.8, 0.9 and 0.9 lower rates of foot examination, retinal eye examination, and hemoglobin A1c measurement respectively to monitor T2D complications compared to non-Hispanic whites.

**Slide 11:** Statistics: Prevalence of Type 2 Diabetes among Hispanic Subgroups

*The facilitator will discuss the prevalence of T2D among different Hispanic groups and nationalities.*

- Puerto Ricans (~13.3% higher risk of developing T2D)
- Mexicans (~11.1% higher risk of developing T2D)
- Dominicans (~9.4% higher risk of developing T2D)
- Cubans (~9.0% higher risk of developing T2D)
- Central Americans (~7.3% higher risk of developing T2D)
- South Americans (~5.0 higher risk of developing T2D)
- Other Hispanics (~7.2 higher risk of developing T2D)

**Slide 12:** Why do Diabetes Rates Differ Among Hispanic Subgroups?

*The facilitator will discuss the differences in type 2 diabetes prevalence among Hispanic subgroups, which result from a mix of genetic, lifestyle, socioeconomic, and cultural factors.*

- Genetics
- Dietary Habits
- Socioeconomic Status
- Cultural Practices

**Slide 13:** Type 2 Diabetes Genetic Susceptibility

*The facilitator will discuss several genetic variants that have been identified, using whole-exome sequencing analysis, as possible culprits for the higher genetic susceptibility to T2D that is seen in Hispanic populations.*

- SLC16A11 variant:
  - expressed in the liver
  - controls lipid metabolism
  - variant has a strong association with type 2 diabetes development
  - First locus specific to Mexicans and Latin Americans

**Slide 14:** Type 2 Diabetes Genetic Susceptibility (cont.)

*The facilitator will discuss several genetic variants that have been identified, using whole-exome sequencing analysis, as possible culprits for the higher genetic susceptibility to T2D that is seen in Hispanic populations.*

- HNF1A (hepatic nuclear factor 1 homeobox A gene) variant:
  - Variant causes amino acid change from glutamate to lysine in the HNF1A gene
  - Causes maturity onset of diabetes of the young
  - 5-fold increased risk of developing type 2 diabetes
  - Individuals with this mutation respond better to sulfonylurea therapy compared to insulin or metformin treatment

**Slide 15:** Type 2 Diabetes Genetic Susceptibility (cont.)

*The facilitator will discuss several genetic variants that have been identified, using whole-exome sequencing analysis, as possible culprits for the higher genetic susceptibility to T2D that is seen in Hispanic populations.*

- IGF 2 (insulin-like growth factor 2) variant:
  - Genetic variant of IGF 2 gene is associated with 20% reduced risk of type 2 diabetes
  - Loss-of-function variant that offers protective genetic effects
  - Absence of the variant is associated with increased incidence of type 2 diabetes

**Slide 16:** What are some metabolic factors that contribute to type 2 diabetes among Hispanics patients?

*The facilitator will explain the metabolic and biological factors that contribute to the development of T2D in Hispanics.*

- Obesity:
  - How it contributes: Excess body fat—especially visceral (abdominal) fat—leads to chronic low-grade inflammation and hormonal imbalances that interfere with insulin action.
  - Relevance to Hispanics: Hispanic populations in the U.S. tend to have higher rates of obesity due to a mix of genetic susceptibility, limited access to healthy foods, lower physical activity levels, and socioeconomic disparities. This increases their risk for insulin resistance and eventually T2D.
- Insulin resistance:
  - How it contributes: Insulin resistance occurs when cells in muscles, fat, and the liver don’t respond well to insulin and can't use glucose for energy effectively. As a result, the pancreas makes more insulin, which over time can lead to beta cell burnout.
  - Relevance to Hispanics: Insulin resistance is often more prevalent and occurs at lower BMI thresholds in Hispanics compared to other populations. This may be due to genetic factors (e.g., variants in genes) and metabolic differences (such as higher liver fat content).
- Beta cell dysfunction in the pancreas:
  - How it contributes: In T2D, pancreatic beta cells gradually lose their ability to produce enough insulin. This dysfunction, often due to prolonged insulin resistance and inflammation, is a critical turning point from prediabetes to full-blown diabetes.
  - Relevance to Hispanics: Research shows that Hispanic individuals may have an inherent predisposition to beta cell dysfunction, potentially due to both genetic and early-life environmental exposures (e.g., early obesity).
- Gut microbial dysbiosis:
- How it contributes: An imbalance in gut bacteria can affect how the body metabolizes glucose and fat, promotes systemic inflammation, and contributes to insulin resistance.
- Relevance to Hispanics: Diets high in processed carbohydrates, sugars, and fats (common in low-resource areas) can negatively alter the gut microbiota. Some studies suggest Hispanics may show gut microbiome patterns associated with higher metabolic disease risk.

**Case Discussions**

*Facilitators should review cases and questions in advance and familiarize themselves with the suggested responses and reflection prompts provided below. While exact wording from learners will vary, the goal is to guide discussion towards recognition of structural barriers, patient-centered communication, and realistic care planning. When discussing lifestyle recommendations (diet, exercise, medication adherence), facilitators should explicitly prompt learners to consider environmental and structural barriers. Examples include:*

- *Neighborhood safety limiting outdoor exercise*
- *Lack of access to affordable fresh foods*
- *Seasonal or climate-related barriers to physical activity*
- *Work schedules, caregiving responsibilities, or multiple jobs*
- *Transportation barriers*
- *Language challenges*

*Facilitators should emphasize that effective diabetes care require adapting recommendations to patients’ environments rather than expecting individual behavior change alone.*

**Slide 17:** Case 1 Discussion

*The audience will be divided into small groups and 5 minutes will be given to read the case and reflect on the questions provided at the end. Then, the facilitator will lead a discussion and reflection session, where the questions and major points will be addressed and debated as a group. Facilitators should emphasize that there if no single “correct” answer to the questions. The goal is to guide learners towards patient-centered, structurally informed decision-making using principles of cultural humility rather than assumptions about cultural norms.*

- Questions:
  1. According to the results of her genetic testing, which medication should her PCP recommend?
- Elena tested positive for a variant of the HNF1A gene, which is associated with a form of diabetes that often responds very well to low-dose sulfonylureas, a class of oral diabetes medications that stimulate insulin secretion.
- Recommended medication:
  Sulfonylureas such as glipizide or glyburide are usually the first-line treatment, often preferable over insulin or metformin in these cases.

**Facilitator Teaching Point**: Facilitators should highlight the importance of shared decision-making and patient education when discussing pharmacologic options, particularly when genetic information if used to guide therapy.

**Ideal Response: Learners should recognize that Elena’s HNF1A variant is associated with a form of diabetes that often responds well to low-dose sulfonylureas. An appropriate recommendation would include sulfonylureas (e.g., glipizide), while also emphasizing patient education, monitoring for hypoglycemia, and shared decision-making. Facilitators should reinforce that genetic-guided therapy must always be contextualized within the patient’s preferences, access, and understanding.**

- 1. What cultural factors could play a role in Elena’s decisions about management and treatment of her condition?
     - Belief in traditional or natural medicine: Elena expresses a strong preference for natural remedies and distrusts Western medicine, which may lead to poor adherence to prescribed treatments.
     - Familial influence: Her family's health beliefs and experiences (many relatives with diabetes using natural remedies) may reinforce her skepticism toward standard medical care.
     - Language barriers and health literacy: As a recent immigrant, Elena may face language difficulties or have limited familiarity with the U.S. healthcare system, affecting communication and understanding.
     - Mistrust of the medical system: Some Hispanic communities have historical or systemic reasons for distrust, which can affect engagement with care.
     - Cultural perception of illness: In some cultures, chronic illnesses like diabetes may carry stigma or be seen as a personal failing, impacting how openly patients talk about their condition or seek help.

**Expanded Reflection Prompt:** **Facilitators should encourage learners to avoid framing these factors as “noncompliance” and instead explore how prior healthcare experiences, structural inequities, and communication barriers may shape patient preferences and trust.**

**Ideal Response: Learners should identify multiple intersecting factors, including belief in traditional medicine, family influence, language barriers, health literacy, and mistrust of healthcare systems. Facilitators should guide learners away from stereotyping and toward understanding how lived experiences, immigration status, and prior healthcare interactions influence trust and adherence.**

- 1. How can healthcare providers effectively collaborate with patients like Elena to develop a comprehensive and culturally sensitive diabetes management plan?

#### **Build trust and rapport**

- Acknowledge and respect her belief in natural medicine without judgment.
- Ask open-ended questions about her remedies and integrate her perspectives into the discussion.

#### **Practice culturally sensitive communication**

- Use **trained medical interpreters** if language is a barrier.
- Provide **education materials in Spanish** and at an appropriate literacy level.
- Consider cultural norms around family involvement

#### **Integrate traditional practices when possible**

- Ask about specific natural remedies and check for any **interactions with prescribed medications.**
- Offer to **coordinate care with her traditional healer**, if feasible, to promote shared understanding and alignment.

#### **Empower with education**

- Use culturally relevant metaphors or visuals to explain diabetes
- Emphasize the **long-term benefits of combining natural and Western approaches** when appropriate.

#### **Facilitate continuity of care**

- Help her access a **bilingual community health worker** or **diabetes support group.**
- Ensure easy follow-up access to a **PCP who is culturally competent** and possibly of similar background.

**Ideal Teaching Emphasis: Facilitators should reinforce that cultural humility involves ongoing learning, self-reflection, and adapting care plans to patient values rather than attempting to “master” cultural traits.**

**Ideal Response: An effective approach includes building rapport, validating Elena’s beliefs, using clear and culturally appropriate education, incorporating safe traditional practices when possible, and engaging family or community resources. Facilitators should emphasize cultural humility, ongoing dialogue, and flexibility rather than rigid treatment plans.**

**Barriers to Implementation: Facilitators should prompt learners to discuss barriers such as limited access to culturally concordant providers, time constraints during clinic visits, lack of bilingual educational materials, and fear or mistrust of medications. Learners should brainstorm strategies to mitigate these barriers (e.g., community health workers, longer follow-up intervals, or bilingual diabetes educators).**

**Slide 18:** Case 2 Discussion

*The audience will be divided into small groups and 5 minutes will be given to read the case and reflect on the questions provided at the end. Then, the facilitator will lead a discussion and reflection session, where the questions and major points will be addressed and debated as a group.* *This case is intended to highlight how emotional health, language access, and social isolation intersect with chronic disease management in older adults.*

- Questions

1. Identify which barriers presented in the case would interfere with Mrs. Rodriguez’s proper management and treatment of this condition:

- Language barrier: The physician is not fluent in Spanish, and the usual medical assistant (translator) was absent. Use of Google Translate is unreliable for nuanced medical communication.
- Health literacy: Mrs. Rodriguez did not fully understand the new insulin regimen, but left with the prescription without clarification.
- Cultural factors: She may defer to authority figures and avoid questioning providers out of respect or discomfort.
- Emotional and social isolation: The death of her husband and limited daily interactions may reduce her motivation and support for managing her condition.
- Dietary habits: Her preferred foods are high in carbohydrates and sugars, which can worsen blood glucose control.

**Ideal Response: Learners should identify language barriers, low health literacy, emotional distress, social isolation, cultural norms discouraging questioning authority, and dietary challenges. Facilitators should highlight that these barriers are systemic rather than individual failures.**

2. How has the loss of her husband affected Mrs. Rodriguez’s overall well-being and management of her diabetes? What actions should she take regarding her mental health?

- Emotional impact: The loss of her husband has left her lonely and grieving, affecting her mood and possibly leading to depression, which is common among older adults with chronic illness and recent bereavement.
- Routine disruption: The loss of daily companionship, including walking together, may reduce her physical activity and emotional resilience.
- Actions to take:
  - Mental health referral: A referral to a Spanish-speaking therapist or counselor familiar with geriatric or grief support.
  - Community resources: Participation in grief support groups (ideally culturally relevant) or senior centers that offer social engagement.
  - Spiritual support: Encouragement to speak with a trusted priest or faith leader, given her regular church attendance.

**Ideal Response: Learners should recognize that grief can worsen glycemic control through depression, decreased motivation, disrupted routines, and reduced physical activity. Appropriate responses include mental health referrals, grief counseling, social engagement, and leveraging community or faith-based resources.**

3. What are some communication strategies that the healthcare provider can use to improve Mrs. Rodriguez’s outcome?

- Use professional interpreters: Always ensure access to trained medical interpreters (in-person or via phone/video) rather than relying on Google Translate or untrained staff.
- Use the "teach-back" method: Ask Mrs. Rodriguez to repeat in her own words what the treatment plan is to ensure understanding.
- Cultural humility and respect: Acknowledge her cultural background, and ask open-ended questions to understand her preferences and beliefs.
- Provide written instructions in Spanish: This can include visuals for insulin use, diet suggestions, and appointment reminders.
- Involve a family member: Many Hispanic older adults rely on family for support with medical care.

**Ideal Response: Effective strategies include professional interpreters, teach-back methods, culturally respectful communication, simplified written materials in Spanish, and involving trusted family members. Facilitators should stress that communication quality directly impacts safety and adherence.**

4. Why might Mrs. Rodriguez have felt embarrassed to ask her primary care physician to clarify her doubts or concerns?

- Cultural norms of respect: In Hispanic cultures, older patients may avoid questioning authority figures, including doctors, as a sign of respect.
- Language insecurity: She may have felt vulnerable or self-conscious about her limited English proficiency.
- Fear of being a burden: Older adults sometimes avoid asking questions to avoid seeming "difficult"
- Emotional fatigue: Grief and isolation may have made her less assertive in her self-care or advocacy.

5. What lifestyle recommendations should be made to Mrs. Rodriguez by her healthcare provider?

The provider should tailor recommendations to her culture and preferences:

- Dietary changes:
  - Encourage moderation, not elimination, of cultural foods (e.g., smaller portions of horchata or use sugar-free options).
  - Recommend balanced meals with lean proteins, non-starchy vegetables, and low-glycemic carbs.
  - Provide a culturally appropriate diabetes meal plan in Spanish.
- Physical activity:
  - Encourage resuming daily walks, possibly with a friend, neighbor, or community group.
- Routine and structure:
  - Reinforce the value of her daily routines (dog walks, church) as stabilizing habits for mental and physical health.
- Glucose monitoring and medication adherence:
  - Train her in insulin administration using clear visuals and simple Spanish explanations.
  - Set up follow-up with a diabetes educator or nurse fluent in Spanish.

**Facilitator Teaching Point: Learners should be encouraged to adapt recommendations (e.g., chair exercises at home, culturally appropriate low-cost dietary substitutions, church-based walking groups) rather than offering generic advice. Facilitators should prompt learners to consider real-world barriers that may limit implementation of lifestyle recommendations, such as:**

- **Unsafe neighborhoods limiting outdoor exercise**
- **Limited access to indoor exercise facilities during extreme weather**
- **Fixed income affecting food choices**
- **Transportation barriers**
- **Grief or depression reducing motivation**

**Ideal Response: Recommendations should be culturally tailored, realistic, and flexible, focusing on gradual dietary changes, low-impact physical activity, and reinforcement of existing routines rather than complete lifestyle overhauls.**

6. What role can religion play in Mrs. Rodriguez’s health?

- Spiritual resilience: Her daily mass attendance suggests strong faith, which may provide comfort, hope, and coping mechanisms.
- Community support: Her church may offer social engagement, health fairs, or caregiving networks.
- Faith leaders as allies: Involving clergy in health promotion can reinforce positive health behaviors if aligned with her beliefs.

**Slide 19:** Case 3 Discussion

*The audience will be divided into small groups and 5 minutes will be given to read the case and reflect on the questions provided at the end. Then, the facilitator will lead a discussion and reflection session, where the questions and major points will be addressed and debated as a group.* *This case is designed to emphasize how socioeconomic constraints and family responsibilities influence diabetes prevention and management at the household level.*

- Questions

1. Identify which factors presented in the case would interfere with Alejandro’s proper management and treatment of this condition:

- Workload and time constraints: Working two jobs, seven days a week leaves little time for physical activity, food preparation, or rest.
- Irregular eating habits: He eats whatever is quick and accessible, which likely includes processed, high-carb, high-fat foods that worsen glycemic control.
- Financial stress: Being the sole income earner for a family of six adds economic pressure, possibly affecting food choices, medication adherence, or doctor visits.
- Caregiving responsibilities: He assists with childcare and household duties, limiting time for self-care.
- Communication barriers: He nods in agreement during visits, but does not disclose his real-life challenges, likely out of shame, cultural norms, or fear of disappointing his provider.

**Ideal Response: Learners should identify time constraints, financial stress, lack of insurance, caregiving demands, irregular meals, stress, and limited social support. Facilitators should emphasize the cumulative burden of social determinants on chronic disease risk.**

2. Identify which factors presented in the case would interfere with Rosa’s proper management and treatment of this condition:

- Lack of health insurance: She may delay or avoid medical care, screenings, and lab work due to cost.
- Time and stress: As a stay-at-home caregiver for four children while also attending school, her stress and time demands are high.
- Limited support system: The couple has no extended family nearby, leading to increased isolation and reduced backup for managing stress and health.
- Economic dependence: With no income and limited access to healthcare, she may struggle to access medications, diabetes prevention programs, or counseling.

**Ideal Response: Learners should identify time constraints, financial stress, lack of insurance, caregiving demands, irregular meals, stress, and limited social support. Facilitators should emphasize the cumulative burden of social determinants on chronic disease risk.**

3. What role can education play in Rosa’s health?

- Health literacy: Understanding prediabetes, nutrition, and physical activity can empower her to make small, meaningful changes for herself and her family.
- Family influence: As the primary caregiver, Rosa’s knowledge can shape the entire household’s health behaviors (meal planning, limiting sugary drinks, encouraging walks).
- Use of online tools: Since she’s pursuing a degree online, she likely has digital access and skills to benefit from virtual diabetes education, cooking classes, or support groups.

4. What roles can health insurance play in Rosa’s health?

- Preventive care: Screenings (A1C, cholesterol, blood pressure), routine check-ups, and early intervention can help prevent the progression to full diabetes.
- Access to medications and services: Coverage can enable access to nutritional counseling, medications like metformin, and lab tests.
- Mental health care: She may benefit from counseling to cope with caregiver stress
- Chronic disease prevention programs: Insurance can facilitate enrollment in lifestyle-change programs

5. What recommendations should be made to Alejandro and Rosa to improve their health?

For Alejandro:

- Nutrition: Teach quick, healthy meal options and portable snacks (e.g., nuts, low-sugar yogurt, whole grain wraps).
- Set small goals: 10-minute walks after work or simple stretching with kids can add up.
- Involve the whole family: Turn physical activity into family playtime (e.g., walking to the park, dancing).
- Connect to community health programs: Refer to local clinics or community health workers for culturally tailored support.

For Rosa:

- Lifestyle education: Recommend free or low-cost prediabetes programs (some online in Spanish).
- Support group connection: Look for virtual support groups for moms or students dealing with chronic conditions.
- Explore coverage options: Help her enroll in health insurance through Medicaid, ACA marketplace, etc.

For both:

- Culturally appropriate guidance: Provide a bilingual diabetes educator or handouts in Spanish with traditional food modifications.
- Family-based care approach: Encourage a joint plan that treats both partners’ health challenges as shared goals.

**Expanded Structural Teaching Point: Facilitators should emphasize that recommendations must account for time, financial constraints, and caregiving demands, and should focus on small, sustainable changes rather than idealized lifestyle goals.**

**Ideal Response: Recommendations should prioritize small, achievable changes such as brief bouts of physical activity, family-based meal planning, culturally appropriate nutrition education, and connection to community or online resources. Facilitators should stress sustainability over perfection.**

**Slide 20:** Socioeconomic Factors Contributing to the Development of Type 2 Diabetes among Hispanic Patients

*The facilitator will summarize examples of socioeconomic factors that contribute to the incidence of T2D among Hispanics (already discussed in cases).*

- High school and college education.
- Poverty rates.
- Unemployment
- Lack of health insurance

**Slide 21:** Lifestyle and Cultural Factors Contributing to the Development of Type 2 Diabetes among Hispanic Patients

*The facilitator will summarize examples of lifestyle and cultural factors that contribute to the incidence of T2D among Hispanics (already discussed in cases).*

- Acculturation: shift toward processed foods, sugary beverages, and modified traditional recipes.
- Nutritional preferences: high-carbohydrate staple foods and calorie-dense cultural meals.
- Physical activity: limited time or safe spaces for regular exercise
- Body image: cultural acceptance of larger body size reducing weight-related concerns
- Family support: household norms influencing diet and lifestyle change
- Health literacy: limited understanding of diabetes, nutritional labels, or complications
- Personal and cultural beliefs: fatalism due to family history; use of home remedies
- Language: limited English proficiency affecting care understanding
- Religion: practices or faith-based health decisions impacting management

**Slide 22:** Treatment and Management Programs for Hispanics

*The facilitator will go over the current T2D treatment and management programs tailored to Hispanics and other minorities.* *Facilitators should explicitly connect these programs to the barriers identified in the cases, highlighting how community-based, peer-led, and culturally responsive models address challenges such as transportation, trust, language, and affordability.*

- The Diabetes Assessment, Nursing, Nutrition, and Dental Evaluation Project
  - Culturally specific diabetes education program on dietary patterns and nutrient composition in men and women with type 2 diabetes
- Project Dulce
  - To improve the quality of care, quality of life, and health among uninsured, low-income minority patients with diabetes. This project was culturally appropriate, community-based, with a nurse case management/peer education diabetes care model, which consisted of a clinical care component and a health and education component
- The Starr County Health Initiative
  - Designed to address the unique needs of the MA population, culturally appropriate regarding language, diet, family participation, and health beliefs. The program included 52 contact hours distributed in weekly 2-hour education sessions for 3 months in nutrition, exercise, and self-monitoring of blood glucose and 14 biweekly 2-hour support group sessions to promote behavior changes and food preparation demonstration
- Tomando Control de su Salud
- Intervention consisted of 14-hour education administered in 2.5-hour sessions over 6 weeks. The topics included in the sessions were fitness, healthy eating, action planning, relaxation, depression, family relationships, and medications

**Slide 23:** Treatment and Management Programs for Hispanics (cont.)

*The facilitator will go over the current T2D treatment and management programs tailored to Hispanics and other minorities. Facilitators should explicitly connect these programs to the barriers identified in the cases, highlighting how community-based, peer-led, and culturally responsive models address challenges such as transportation, trust, language, and affordability.*

- **Tomando control**
  - A program, tested in a pilot study to assess the feasibility, acceptability, and efficacy of a culturally appropriate and culturally relevant cognitive-behavioral diabetes self-care education program for Hispanic Americans with type 2 diabetes
- **Banister et al**
  - An intervention implemented by Banister et al was conducted in Hispanic and African American individuals with T2D at a community clinic in Texas to assess the effectiveness of a diabetes self-management training program.
- **Rosa’s Story**
  - An education tool developed by the Latino Diabetes Initiative at the Joslin Diabetes Center, represents a unique approach to culturally competent diabetes education for Spanish-speaking Latinos at risk and living with type 2 diabetes
- **Puentes hacia una major vida**
  - A program where volunteer peer leaders were used to provide support for patients with type 2 diabetes through telephone contact, in-person, individual, and group sessions

**Slide 24:** Treatment and Management Programs for Hispanics (cont.)

*The facilitator will explain the rationale for including each program, highlight how they address specific cultural or structural barriers impacting Hispanic populations, and suggest how healthcare providers can adopt elements of these programs or connect patients to similar local resources nationwide.*

- These culturally tailored diabetes programs serve as practical examples of how interventions designed specifically for Hispanic and minority populations can address unique cultural, socioeconomic, and behavioral factors that influence diabetes management and outcomes.
- The goal is to raise awareness among healthcare providers and trainees about successful models of care that incorporate cultural competence and community-based support.
- Many of these programs have documented improvements in diabetes-related outcomes, such as better glycemic control, improved self-management behaviors, and increased patient engagement and satisfaction.
- While some programs are region-specific, many serve as models that providers anywhere in the country can learn from.

**Slide 25:** Type 2 Diabtes

*The facilitator will provide a quick summary of the major factors that contribute to the development of T2D in Hispanic populations.* *Facilitators should reinforce that improving diabetes outcomes in Hispanic populations requires addressing upstream structural determinants alongside biological risk factors.*

- Genes (SLC16A11, HNF1A, IGF2, CKN2A, ABCA1, TCF7L2, ATPVIH)
- Socioeconomic (education, poverty, health insurance)
- Environmental (physical activity, diet, microbiome, acculturation, body image)
- Metabolic (obesity, inflammation, endothelial dysfunction, insulin resistance, dyslipidemia)

**Slide 26:** Post-Evaluation QR Code

*The facilitator(s) will indicate to the audience to access the post-evaluation using the QR code and allow time to complete it.*

- QR code needs to be created for the post-evaluation.
