# Supplementary material for: Health Disparities Among Hispanic Patients With Type 2 Diabetes in the United States: An Educational Workshop
Source: MedEdPORTAL. 2026 Jul 22;22:11622. doi: 10.15766/mep_2374-8265.11622 (PMC13388404; doi:10.15766/mep_2374-8265.11622)
Supplement: Supplementary file 1 — Presession Evaluation.docxPostsession Evaluation.docxPresentation.pptxFacilitator Guide.docx [file mep_2374-8265.11622-s001.zip › B. Postsession Evaluation.docx]

**“Health Disparities among Hispanics with type 2 diabetes in the United States”**

**Post-Session Evaluation**

1. Complete the following self-assessment

| **Please rate how much CONFIDENCE do you have in your ability to…** | **No Confidence**  **0** | **Low Confidence**  **1** | **Moderate Confidence**  **2** | **High Confidence**  **3** | **Complete Confidence**  **4** |
| --- | --- | --- | --- | --- | --- |
| Describe health disparities among Hispanic patients with type 2 diabetes in the United States. |  |  |  |  |  |
| Elucidate genetic susceptibility and metabolic factors that contribute to the development of type 2 diabetes among Hispanic patients. |  |  |  |  |  |
| Describe cultural, socioeconomic, and lifestyle factors associated with the incidence of type 2 diabetes among Hispanic patients. |  |  |  |  |  |
| Illustrate through cases the need for more inclusive and tailored treatment options, preventive education, lifestyle changes, and health interventions that are necessary to improve patient outcome. |  |  |  |  |  |

1. Which of the following is not likely a risk factor for type 2 diabetes among Hispanic patients living in the United States?
   1. Genetics
   2. Obesity
   3. Lifestyle
   4. Height
   5. Age
   6. Smoking
2. Hispanic patients living in the United States have:
   1. The highest incidence of type 2 diabetes among all ethnicities
   2. The second highest incidence of type 2 diabetes among all ethnicities
   3. The third highest incidence of type 2 diabetes of all ethnicities
   4. The fourth highest incidence of type 2 diabetes among all ethnicities
3. Which of the following statements is false about Hispanic patients living in the United States?
   1. Hispanic adults have more than a 50% chance of developing type 2 diabetes over their lifetime
   2. Hispanics are more likely to develop type 2 diabetes at an older age compared to other ethnicities
   3. Hispanics have a higher risk of developing complications such as kidney failure associated with type 2 diabetes
   4. Hispanics have a higher risk of developing complications such as visual impairments associated with type 2 diabetes
   5. Death rates from type 2 diabetes among Hispanics are higher than those among non-Hispanic white people
4. Among Hispanic patients living in the United States, which group has the lowest rate of type 2 diabetes?
   1. Puerto Ricans
   2. Mexicans
   3. Cubans
   4. Dominicans
   5. Central/South America
5. Which of the following socioeconomic factors does **not** likely contribute to the development of type 2 diabetes among Hispanic patients in the United States?
   1. Population density
   2. Education
   3. Economic status
   4. Unemployment
   5. Health insurance
6. How would you rate this workshop?
   1. Poor
   2. Fair
   3. Good
   4. Excellent
7. What did you like about this workshop?
8. What suggestions do you have to improve this workshop?
9. How would you implement what you learned in this workshop in the future?
